# Boosting peripheral BDNF rescues impaired in vivo axonal transport in CMT2D mice

**DOI:** 10.1172/jci.insight.157191

**Published:** 2023-05-08

**Authors:** James N. Sleigh, David Villarroel-Campos, Sunaina Surana, Tahmina Wickenden, Yao Tong, Rebecca L. Simkin, Jose Norberto S. Vargas, Elena R. Rhymes, Andrew P. Tosolini, Steven J. West, Qian Zhang, Xiang-Lei Yang, Giampietro Schiavo

**Affiliations:** 1 Department of Neuromuscular Diseases and UCL Queen Square Motor Neuron Disease Centre, UCL Queen Square Institute of Neurology, and; 2 UK Dementia Research Institute, University College London (UCL), London, United Kingdom.; 3 Department of Molecular Medicine, The Scripps Research Institute, La Jolla, California, USA.; 4 Sainsbury Wellcome Centre, UCL, London, United Kingdom.

**Keywords:** Neuroscience, Gene therapy, Neurological disorders, Neuromuscular disease

## Abstract

Gain-of-function mutations in the housekeeping gene *GARS1*, which lead to the expression of toxic versions of glycyl-tRNA synthetase (GlyRS), cause the selective motor and sensory pathology characterizing Charcot-Marie-Tooth disease (CMT). Aberrant interactions between GlyRS mutants and different proteins, including neurotrophin receptor tropomyosin receptor kinase receptor B (TrkB), underlie CMT type 2D (CMT2D); however, our pathomechanistic understanding of this untreatable peripheral neuropathy remains incomplete. Through intravital imaging of the sciatic nerve, we show that CMT2D mice displayed early and persistent disturbances in axonal transport of neurotrophin-containing signaling endosomes in vivo. We discovered that brain-derived neurotrophic factor (BDNF)/TrkB impairments correlated with transport disruption and overall CMT2D neuropathology and that inhibition of this pathway at the nerve-muscle interface perturbed endosome transport in wild-type axons. Accordingly, supplementation of muscles with BDNF, but not other neurotrophins, completely restored physiological axonal transport in neuropathic mice. Together, these findings suggest that selectively targeting muscles with BDNF-boosting therapies could represent a viable therapeutic strategy for CMT2D.

## Graphical abstract

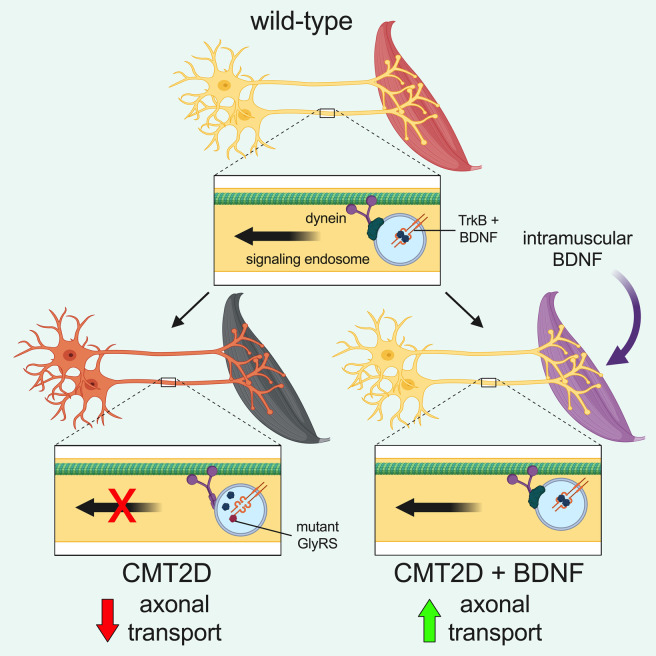

## Introduction

Charcot-Marie-Tooth disease (CMT) is an inherited peripheral nerve disorder resulting in lifelong disability, with no available disease-modifying therapies (1). Patients usually present with muscle weakness and wasting, as well as sensory deficits, in the hands and feet, indicating that motor and sensory neurons with the longest axons are generally the most susceptible to neuropathy. Given that CMT is caused by mutations in more than 90 genes with diverse functions (2), a single unifying pathomechanism is unlikely; nevertheless, compromised in vitro axonal transport has been identified in several CMT subtypes (3, 4) and is hypothesized to be a major driver of axon and synaptic degeneration (5, 6).

CMT type 2D (CMT2D) manifests during adolescence because of dominantly inherited missense mutations in the widely and constitutively expressed *GARS1* gene, which encodes glycyl-tRNA synthetase (GlyRS), an enzyme essential for protein synthesis (7). Whereas the mechanisms causing selective motor and sensory nerve pathology in CMT2D remain incompletely resolved (8), *GARS1* mutations induce a conformational opening in GlyRS (9, 10), exposing common buried surfaces that enable toxic misinteractions with several proteins inside and outside neurons (11–16). Aberrant binding partners include the neuronal and vascular transmembrane receptor protein neuropilin-1 (11) and histone deacetylase HDAC6 (14). Enhanced associations with wild-type GlyRS interactors are also possible. Indeed, recent evidence indicates that mutant GlyRS binds but fails to efficiently release tRNA^Gly^, which leads to ribosome stalling at glycine codons, inhibition of protein synthesis, and activation of the integrated stress response (17, 18). Accordingly, tRNA^Gly^ overexpression rescues neuropathy phenotypes in *Drosophila melanogaster* and mouse models of CMT2D (18). Impaired protein synthesis is thus a major component of *GARS1* neuropathy, as well as other diseases affecting peripheral nerves (19, 20); however, the driver of differential vulnerability in neuropathy across motor and sensory neuron subtypes remains undetermined.

We discovered that CMT2D-causing, but not wild-type, GlyRS interacts with extracellular domains of tropomyosin receptor kinase (Trk) receptors A, B, and C, contributing to the developmental perturbation of sensory neurons in mutant *Gars* mice (13, 21). Via their role as neurotrophin receptors, the Trk receptors are critical to neuronal differentiation and homeostasis (22). Through selective binding and internalization of secreted neurotrophins at axon terminals, Trk receptors are retrogradely transported within signaling endosomes to somas, where they elicit gene transcription events essential for neuronal survival (23, 24). TrkA preferentially binds to nerve growth factor (NGF), TrkB to both brain-derived neurotrophic factor (BDNF) and neurotrophin-4 (NT-4), and TrkC to neurotrophin-3 (NT-3) (22).

In this study, we set out to determine whether in vivo disturbances in axonal transport of neurotrophin-containing signaling endosomes contribute to CMT2D pathology.

## Results

### CMT2D mice display early, persistent perturbations in endosome axonal transport in vivo.

**Figure 1:**
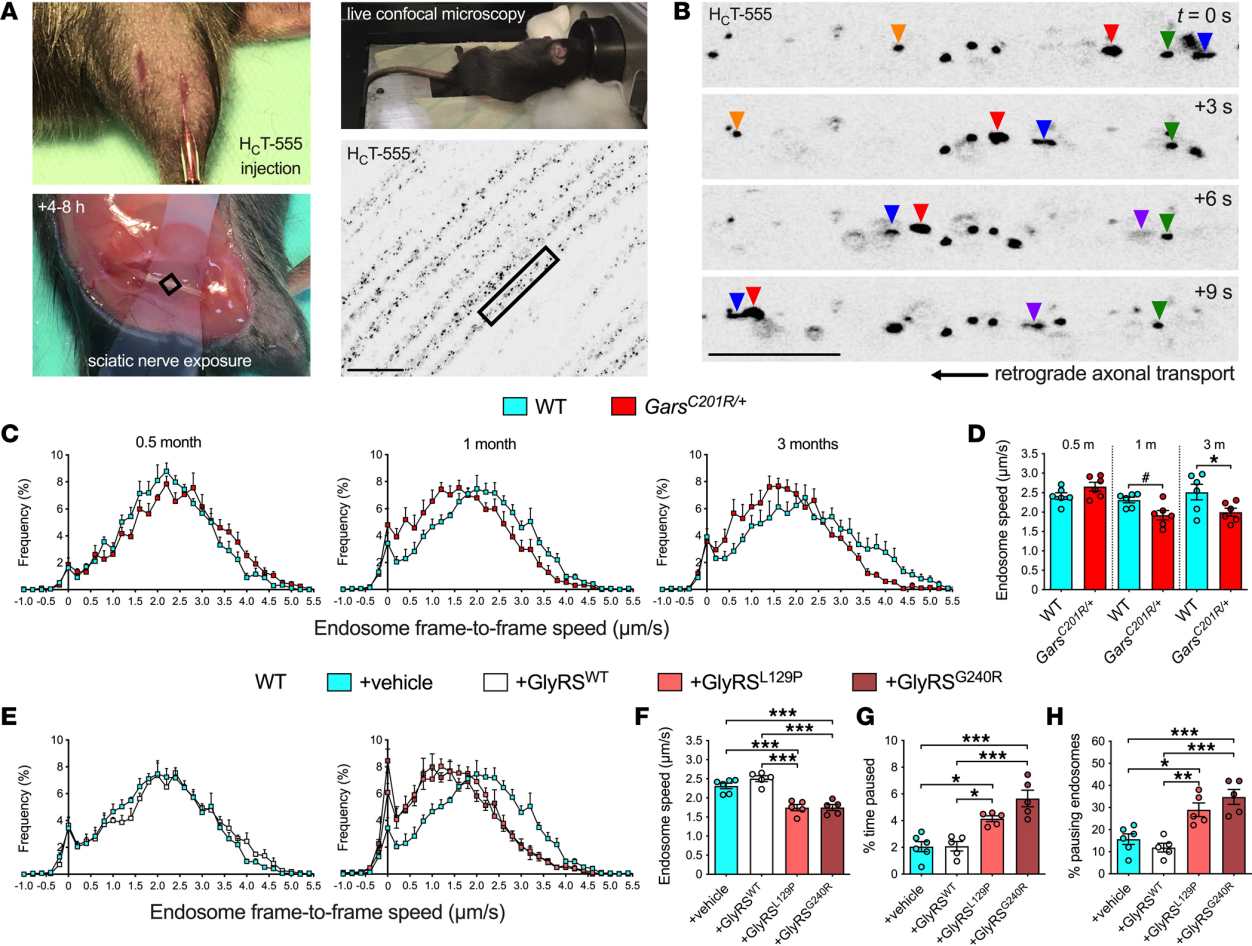
Neuropathy-causing *GARS1* mutations impair retrograde axonal transport of signaling endosomes in vivo. (**A**) Intramuscular injection of a fluorescent fragment of tetanus neurotoxin (H_C_T-555), with subsequent exposure of the sciatic nerve, permits in vivo imaging of signaling endosomes in intact peripheral nerve axons of live, anesthetized mice. (**B**) Retrogradely transported H_C_T-positive endosomes are individually tracked to quantitatively assess their dynamics. Color-coded arrowheads identify 5 endosomes. (**C**) Endosome frame-to-frame speed histograms of wild-type and CMT2D-modeling *Gars^C201R/+^* mice aged 0.5, 1, and 3 months. (**D**) Defective axonal transport manifests in *Gars^C201R/+^* mice between 0.5 and 1 month of age (genotype *P* = 0.033, age *P* = 0.006, interaction *P* = 0.009, 2-way ANOVA). (**E**) Endosome frame-to-frame speed histograms of 1-month-old wild-type mice receiving intramuscular injections of recombinant human wild-type or CMT2D-causing GlyRS. (**F**–**H**) GlyRS^L129P^ and GlyRS^G240R^, but not GlyRS^WT^, cause a cell-nonautonomous decrease in signaling endosome speed (**F**), increased pause time (**G**) and more pausing endosomes (**H**) in healthy axons (**F**–**H**, *P* < 0.001, 1-way ANOVA). For all graphs, *P < 0.05, **P < 0.01, ***P < 0.001, Šídák’s multiple comparisons test; ^#^*P* < 0.05, unpaired *t* test; *n* = 5–6. Scale bars = 20 μm (**A**), 10 μm (**B**). See Supplemental Figure 1.

Injection of a well-characterized atoxic fluorescent fragment of tetanus neurotoxin (H_C_T-555) (25) into distal leg muscles permits in vivo imaging and tracking of signaling endosomes within intact sciatic nerve axons of live, anesthetized mice (Figure 1, A and B) (26–28). By imaging thicker axons only, we have previously shown that transport is assessed in motor rather than sensory neurons (29). H_C_T-555 was therefore injected into wild-type and *Gars^C201R/+^* mice, which carry a toxic gain-of-function mutation in endogenous *Gars* (30). The C201R mutation causes an array of progressive motor and sensory phenotypes modeling CMT2D that present by 1 month of age, as well as nonprogressive neurodevelopmental alterations (13, 21, 30–33). Coinciding with the onset of neuromuscular junction (NMJ) denervation in the most severely affected hind paw muscles (31), *Gars^C201R/+^* mice exhibited an overt decline in in vivo endosome transport speed that manifested between 0.5 and 1 month of age, persisting to at least 3 months (Figure 1, C and D).

To gauge the human relevance of this phenotype, we assessed endosome transport in a second CMT2D mouse model, *Gars*^ΔETAQ/+^, which carries a deletion in endogenous mouse *Gars* modeling a mutation identified in a patient presenting with severe, early-onset neuropathy (34). First, we verified that human GlyRS^ΔETAQ^ aberrantly interacts with the extracellular domain of human TrkB (Supplemental Figure 1, A and B; supplemental material available online with this article; https://doi.org/10.1172/jci.insight.157191DS1) and that the *Gars*^ΔETAQ/+^ strain displays the same perturbation of sensory neuron fate present in other mutant *Gars* alleles (Supplemental Figure 1, C and D), i.e., decrease in neurofilament 200 and increase in peripherin levels in lumbar dorsal root ganglia (DRG) (13, 21). Analyzing signaling endosome dynamics, we found that 1-month-old *Gars*^ΔETAQ/+^ mice also showed impaired in vivo axonal transport early in disease and to a similar extent as *Gars^C201R/+^* (Supplemental Figure 1, E–L), indicating that trafficking disruption is a phenotype common to mouse strains modeling CMT2D.

To further link transport disruption to the human neuropathy, we premixed and coinjected H_C_T-555 with purified human wild-type or CMT2D-causing GlyRS protein into distal leg muscles of wild-type mice. GlyRS^L129P^ and GlyRS^G240R^ were selected because of the strong human genetic evidence supporting their causative role in neuropathy (8, 35). We found that both mutants, but not GlyRS^WT^, perturbed transport of signaling endosomes in otherwise-healthy peripheral nerve axons of wild-type animals (Figure 1, E–H). This result indicates that extracellular exposure of motor nerve terminals to mutant GlyRS is sufficient to alter axonal endosome trafficking, suggesting the presence of a cell-nonautonomous component to this phenotype, as previously identified in a *Drosophila* CMT2D model, where muscle-derived mutant GlyRS accumulates at NMJs and coincides with motor neuron degeneration (12, 15).

Together, these findings indicate that neuropathy-causing *GARS1* mutations impair axonal transport of neurotrophin-containing signaling endosomes in motor neurons in vivo and that human mutant GlyRS injected into muscle can induce this phenotype in wild-type mice.

### Muscle TrkB levels correlate with denervation in CMT2D.

We next sought to identify whether alterations in the BDNF/TrkB pathway correlate with neuropathy in CMT2D. In *Gars^C201R/+^* mice we have identified a spectrum of vulnerability to NMJ denervation across 5 whole-mount muscles with diverse morphological and functional properties (36); transversus abdominis (TVA), epitrochleoanconeus (ETA), forelimb lumbrical, hind limb lumbrical, and flexor digitorum brevis (FDB) muscles display 0%, 0%, 2.5%, 11.4%, and 20.3% complete denervation at 3 months, respectively (33). We determined that developmental demands and axon length are linked to the loss of neuromuscular connectivity; however, these features do not fully determine susceptibility to disease (33).

**Figure 2:**
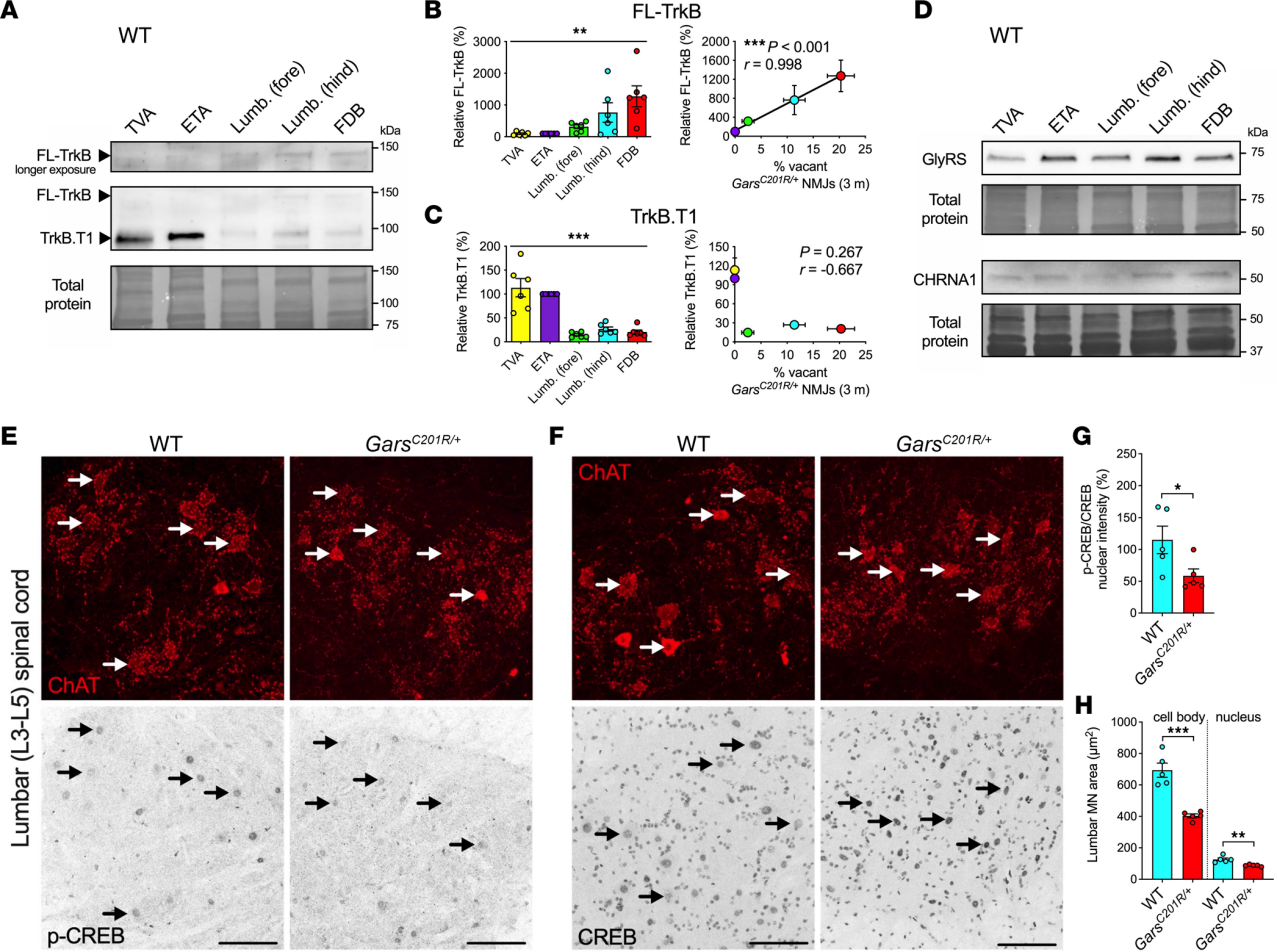
Dysfunctional BDNF/TrkB signaling correlates with CMT2D pathology. (**A**) Representative Western blots of lysates from 5 wild-type muscles, probed with anti-TrkB, displaying a spectrum of vulnerability to NMJ denervation in *Gars^C201R/+^*, with the transversus abdominis (TVA) and epitrochleoanconeus (ETA) being least impacted and the flexor digitorum brevis (FDB) displaying the greatest degeneration (33). (**B** and **C**) Levels of FL-TrkB (**P = 0.001, 1-way ANOVA) and TrkB.T1 (***P < 0.001, Kruskal-Wallis test) differ between muscles. FL-TrkB (***P < 0.001, Pearson’s product moment correlation), but not TrkB.T1 (*P* = 0.267 Spearman’s rank correlation), positively correlates with CMT2D denervation. (**D**) Representative Western blots of lysates from 5 wild-type muscles, probed with anti-GlyRS and anti-CHRNA (quantified in Supplemental Figure 3, C and D). (**E** and **F**) Representative collapsed *Z*-stack confocal images of lumbar spinal cord ventral horns from wild-type and *Gars^C201R/+^* mice stained for ChAT and p-CREB or CREB. Lower panels: inverted fluorescence images. Arrows: motor neuron nuclei. Scale bars = 100 μm. (**G** and **H**) *Gars^C201R/+^* lumbar motor neurons display reduced p-CREB activation (**G**) and are smaller than their wild-type counterparts (**H**). *P < 0.05, **P < 0.01, ***P < 0.001 unpaired *t* test. For all graphs, *n* = 5–6. Mice were P77–P83 (**A**–**D**) and 3 months old (**E**–**H**). See Supplemental Figures 2–5.

We therefore dissected the same 5 muscles from wild-type mice and probed lysates for TrkB (Figure 2, A–C), the neurotrophin receptor most crucial to NMJ function and stability (37). If aberrant interactions between mutant GlyRS and TrkB disrupt BDNF/TrkB signaling, thereby contributing to peripheral denervation, then levels of TrkB, BDNF, and/or GlyRS within muscles and NMJs may correlate with the degree of CMT2D pathology. Wild-type, rather than *Gars^C201R/+^*, muscles were analyzed so that baseline protein availability could be determined without interference from the differential NMJ denervation that occurs in CMT2D mice (33), which probably causes the reduced full-length TrkB (FL-TrkB) levels identified in *Gars^C201R/+^* muscles (Supplemental Figure 2). Western blotting of the thin whole-mount muscles is technically challenging because of the low protein yield, especially from the lumbrical and FDB muscles. Nevertheless, we identified consistent differences between wild-type muscles in the availability of FL-TrkB and truncated TrkB.T1 (Figure 2, A–C). Levels of FL-TrkB, which is fundamental for pro-survival signaling, correlated with *Gars^C201R/+^* denervation (Figure 2B), whereas levels of TrkB.T1, which lacks the essential kinase domain, did not (Figure 2C).

Similar Western blot analyses were attempted with anti-BDNF antibody, but clear bands representing proBDNF and mature BDNF (mBDNF) at the expected molecular weights of ≈35 and 14 kDa, respectively, were not detected. Nevertheless, performing immunofluorescence analyses, we found that total BDNF levels at the wild-type NMJ inversely correlated with the percentage of partially denervated synapses in CMT2D mice (Supplemental Figure 3, A and B). GlyRS levels did not differ significantly between muscles (Figure 2D and Supplemental Figure 3C), indicating that differences in GlyRS availability are unlikely to be a major driver of NMJ degeneration. Moreover, levels of CHRNA1, an acetylcholine receptor subunit found at the NMJ, were similar across muscles (Figure 2D and Supplemental Figure 3D), indicating that distinctions in number or size of NMJs do not account for the difference in FL-TrkB levels. Notably, there were also no differences in the availability of the motor neuron survival factor glial cell line–derived neurotrophic factor (Supplemental Figure 3, E–G), suggesting that the alterations found in CMT2D are specific for BDNF.

These data support the hypothesis that differences in BDNF-TrkB levels within muscles and at the NMJ may contribute to the selective vulnerability of motor nerve terminals to *GARS1* neuropathy.

### Impaired cAMP response element-binding protein activation and motor neuron size are linked to CMT2D motor degeneration.

The decline in endosome speed in *Gars^C201R/+^* mice will reduce BDNF delivery to motor neuron somas. Provided that endosome flux remains unaffected, the observed 16.8% and 20.5% reductions in speed at 1 and 3 months (Figure 1), respectively, would equate to more than an extra day per week being needed to deliver the same amount of neurotrophins to the spinal cord of CMT2D mice. We hypothesized that this decrease would therefore cause dampened activation of signaling pathways that maintain neuronal health and survival, which could contribute to the slowly progressive motor and sensory neuron degeneration in CMT2D.

To assess this, lumbar spinal cords were dissected from 3-month-old mice and stained for phosphorylated and total cAMP response element-binding protein (CREB) (Figure 2, E and F), a pro-survival transcription factor, which is activated in response to BDNF (38). Although differences between genotypes in nuclear phosphorylated CREB (p-CREB) or CREB levels did not reach significance in choline acetyltransferase–positive (ChAT-positive) motor neurons (Supplemental Figure 4A), CREB activation, as indicated by the ratio between p-CREB and CREB levels, was constrained in CMT2D mice (Figure 2G). Moreover, although no loss of these hind limb–innervating lumbar motor neurons was found (Supplemental Figure 4B), we identified a clear reduction in the area of *Gars^C201R/+^* motor neuron cell bodies and nuclei (Figure 2H).

A decrease in motor neuron size could result from switching of α motor neuron subtype from large, fast-twitch fatigable to small, slow-twitch fatigue-resistant motor neurons or even γ motor neurons. However, we have previously shown lack of change in proportion of α and γ motor neurons in *Gars^C201R/+^* spinal cords at 1 month (13), indicating that this is not the cause of the reduced lumbar motor neuron area. We thus attempted spinal cord staining for markers of different α motor neuron subtypes (e.g., chondrolectin, estrogen-related receptor β, and matrix metalloproteinase-9) but were unsuccessful. Muscle fiber and α motor neuron subtypes are closely related (39), and denervation can induce changes in motor unit identity (40); thus, by staining for myosin heavy chain (MHC) isoforms, we assessed the proportions of muscle fiber types in transverse sections of tibialis anterior muscles from 3-month-old wild-type and *Gars^C201R/+^* mice (Supplemental Figure 5A). The tibialis anterior is known to display weakness and NMJ denervation at this age in this mouse strain (30). Type I fibers are the smallest and largely innervated by slow-twitch fatigue-resistant α motor neurons, whereas type II fibers are larger (increasing in size from IIa to IIx to IIb) and innervated by either fast-twitch fatigue-resistant (IIa/IIx) or fast-twitch fatigable α motor neurons (IIb/IIx) (39).

We first determined that there were no differences between genotypes in the cross-sectional area of each myofiber subtype (Supplemental Figure 5B). We then showed that *Gars^C201R/+^* mice possessed a lower percentage of MHC type IIb fibers and higher percentages of type IIx and type I fibers (Supplemental Figure 5C). However, by assessing the number of each fiber subtype, we identified that compared with wild-type, *Gars^C201R/+^* muscles possessed fewer type IIb fibers, similar numbers of type IIx fibers, and more type I fibers (Supplemental Figure 5D); this resulted in a reduction in total myofiber number (Supplemental Figure 5E), which may deplete target-derived BDNF, exacerbating transport disruption.

Together, these data suggest that the tibialis anterior muscle of CMT2D mice displays a selective loss of large, fast-twitch fatigable α motor neurons and an increase in slow-twitch fatigue-resistant α motor neurons, likely due to a combination of fiber loss and subtype switching. Along with impaired neurotrophin signaling and dampened protein synthesis (41), this change in subtype proportions will likely contribute to the reduced size of CMT2D lumbar motor neurons.

Dissimilar to the lumbar motor neurons, upper body–innervating thoracic motor neurons of CMT2D mice showed no difference from control in CREB phosphorylation (Supplemental Figure 4, C–E). Like in the lumbar spinal cord, no loss of thoracic motor neurons was observed (Supplemental Figure 4F); however, in contrast to the lumbar motor neurons, soma and nucleus size were unaffected in thoracic motor neurons (Supplemental Figure 4G), indicating that the reduced area of lumbar motor neurons is not caused by the smaller size of CMT2D mice. This was further corroborated by analyzing motor neuron areas relative to the area of the spinal cord section from which they were measured (data not shown). These collective data show that impairments in CREB activation and motor neuron size are selective and linked to the more severe motor and sensory pathology observed in CMT2D hind limbs (21, 33).

### Reduced endosome adaptor protein levels correlate with CMT2D pathology.

**Figure 3:**
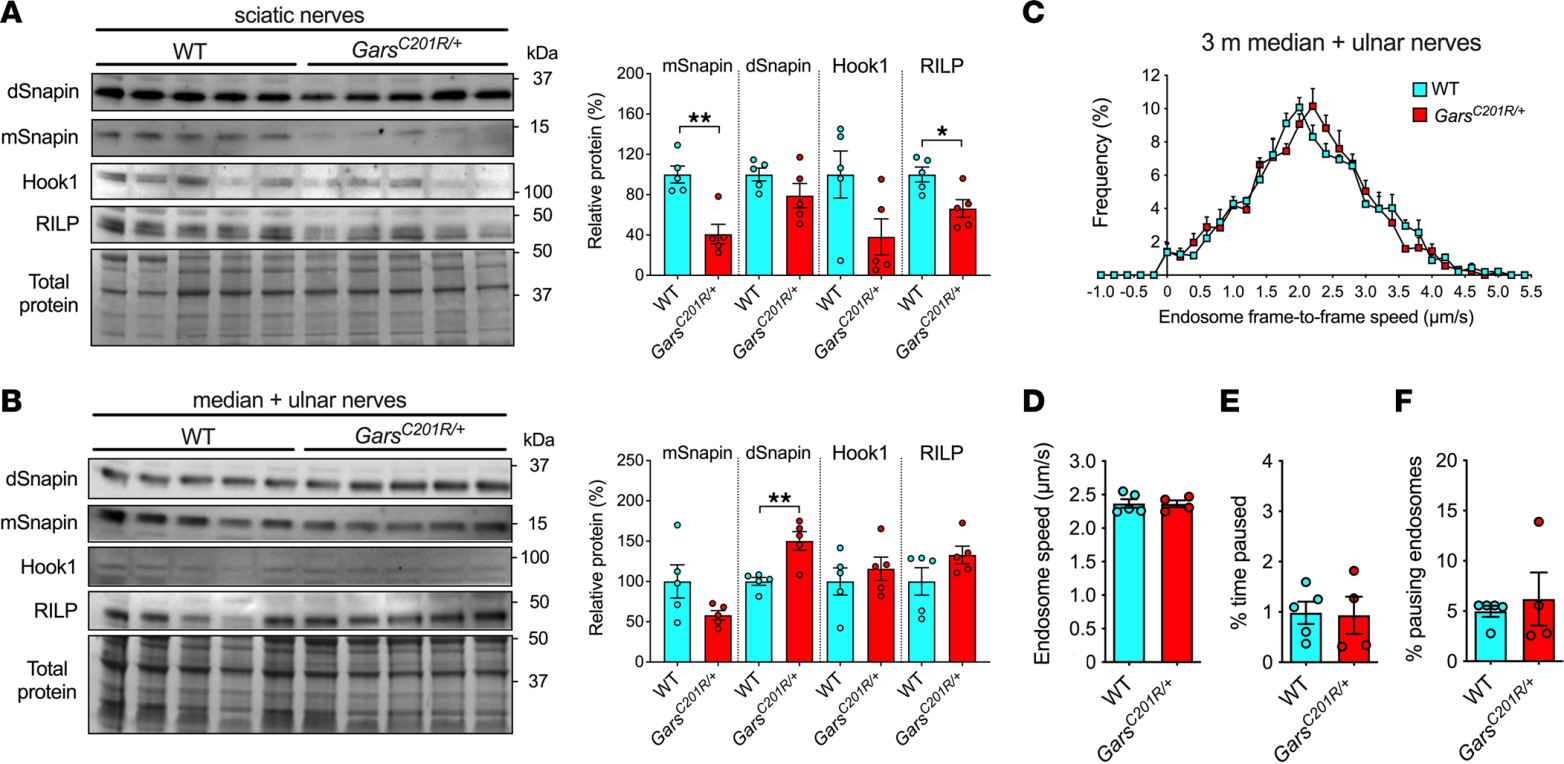
Endosome adaptor protein levels are selectively reduced in hind limb–innervating nerves. (**A**) Western blot of dynein adaptor proteins Snapin, Hook1, and RILP in sciatic nerves (hind limb) from 1-month-old wild-type and *Gars^C201R/+^* mice. Densitometric analyses show reduced levels of monomeric Snapin (mSnapin, **P = 0.002) and RILP (*P = 0.018) in *Gars^C201R/+^* sciatic nerves. (**B**) Western blot of Snapin, Hook1, and RILP in combined median and ulnar nerves (forelimb) from 1-month-old wild-type and *Gars^C201R/+^* mice. Contrasting with sciatic nerves, densitometric analysis identified an increase in dimeric Snapin (dSnapin, **P = 0.004) in median and ulnar nerves. (**C**) Endosome frame-to-frame speed histograms from 3-month-old wild-type and *Gars^C201R/+^* median and ulnar nerves. (**D**–**F**) Signaling endosome transport speed (**D**, *P* = 0.954), pause time (**E**, *P* = 0.910), and pause percentage (**F**, *P* = 0.810, Mann-Whitney *U* test) in forelimb-innervating nerves are unaffected in *Gars^C201R/+^* mice. Genotypes were compared using unpaired *t* tests, unless otherwise stated; *n* = 4–5.

Having assessed distal (NMJs) and proximal (cell bodies) subcellular regions of the motor neuron, we wanted to address whether any axonal disturbances in endosomal transport proteins could be observed in CMT2D mice. Adaptor proteins are required for connecting the retrograde motor cytoplasmic dynein to specific cargoes, such as TrkB-positive signaling endosomes, and their reduced availability could contribute to impaired axonal trafficking (42). To determine whether CMT2D mice display differences in endosome adaptors, we probed lysates from hind limb–innervating sciatic nerves and forelimb-innervating median and ulnar nerves for the established cytoplasmic dynein-binding proteins Snapin (43), Hook1 (44), and RILP (45). In agreement with the peripheral nerve pathology, we found that levels of monomeric Snapin (mSnapin) and RILP were lower in *Gars^C201R/+^* sciatic nerve (Figure 3A), whereas there was no significant downregulation in median and ulnar nerves (Figure 3B), greatly decreasing the possibility of a systemic phenotype.

### Endosome axonal transport is unaffected in forelimb-innervating nerves.

Given that neuromuscular pathology is comparatively mild or absent in CMT2D forelimbs, we adapted our in vivo imaging approach to perform intravital analysis of signaling endosome transport in median and ulnar nerves (46). To target muscles innervated by motor neurons within these nerves, we injected H_C_T-555 into the forepaw of wild-type and *Gars^C201R/+^* mice at 3 months, representing an age at which axonal transport has been defective in CMT2D sciatic nerves for at least 2 months (Figure 1, C and D). Consistent with the neuropathology, there was no difference in signaling endosome dynamics between wild-type and *Gars^C201R/+^* median and ulnar nerves (Figure 3, C–F). Moreover, endosome speeds of both genotypes were similar to those observed in 3-month-old wild-type sciatic nerves (wild-type sciatic 2.51 ± 0.2 µm/s; wild-type median/ulnar 2.37 ± 0.1 µm/s; *Gars^C201R/+^* median/ulnar 2.36 ± 0.1 µm/s; *P* = 0.699, 1-way ANOVA).

In summary, differences in pro-survival FL-TrkB between muscles strictly correlate with NMJ denervation in CMT2D mice. Furthermore, activation of the pro-survival and BDNF-dependent transcription factor CREB is stunted in *Gars^C201R/+^* lumbar motor neurons, and this effect is specific, as it is not observed in thoracic motor neurons. Finally, endosome adaptor proteins critical to axonal transport are selectively downregulated in hind limb– but not forelimb-innervating nerves, correlating with disturbances in endosome axonal transport in motor neurons in vivo. Given the importance of neurotrophin signaling to neuronal health and survival, these phenotypes likely contribute to the selective motor neuropathology observed in CMT2D.

### Altering BDNF/TrkB signaling at healthy nerve terminals impairs endosome transport.

**Figure 4:**
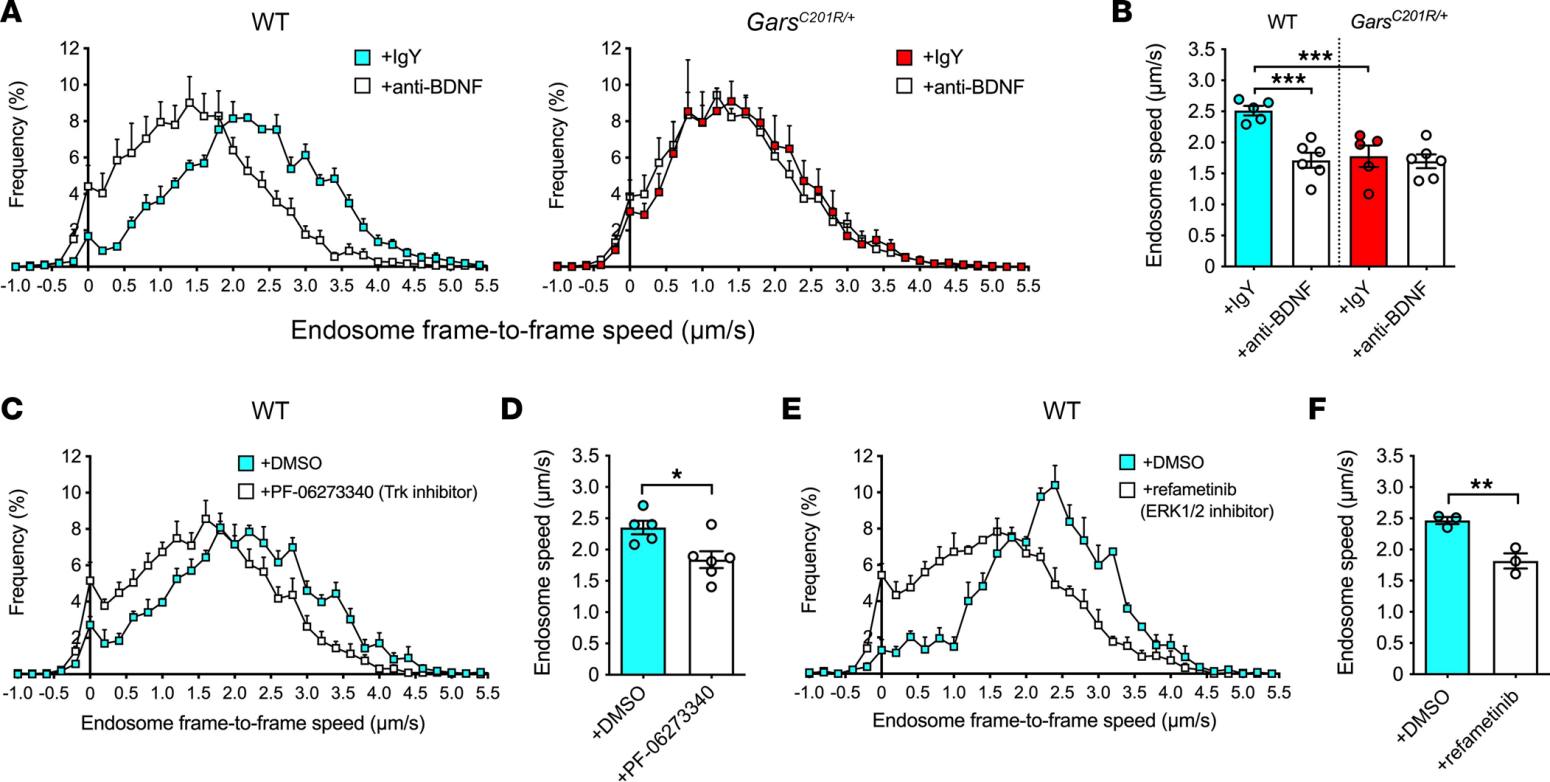
In vivo BDNF depletion and TrkB inhibition impair endosome transport via ERK1/2. (**A**) Endosome frame-to-frame speed histograms of wild-type and *Gars^C201R/+^* mice 4–8 hours posttreatment with intramuscular injections of anti-BDNF or IgY control antibody. (**B**) Anti-BDNF slows wild-type endosome transport yet has no effect on *Gars^C201R/+^* (*P* < 0.001, 1-way ANOVA). ***P < 0.001, Šídák’s multiple comparisons test. *n* = 5–6. (**C** and **D**) Intramuscular injection of 50 nM pan-Trk inhibitor slows signaling endosome transport in wild-type motor axons. *P < 0.05, unpaired *t* test. *n* = 5–6. (**E** and **F**) Intramuscular injection of 50 nM ERK1/2 inhibitor impairs endosome transport in wild-type mice. **P < 0.01, unpaired *t* test. *n* = 3. Mice were 1 month old, except **E** and **F** (P40–P45). See Supplemental Figures 6 and 7.

Our data thus far are commensurate with aberrant mutant GlyRS/TrkB interactions at peripheral nerve terminals underlying impaired BDNF signaling in CMT2D. To test whether dampening BDNF/TrkB signaling at the nerve-muscle interface disrupts endosome transport and explains the neuropathic phenotype we observed in vivo, we coinjected H_C_T-555 with anti-BDNF antibodies into wild-type and *Gars^C201R/+^* leg muscles. This would sequester extracellular BDNF in the vicinity of peripheral nerve terminals and impede signaling through TrkB. While immunoglobulin Y (IgY) control antibodies had no effect on axonal transport, restricting the availability of BDNF at wild-type NMJs resulted in a slowdown in signaling endosome transport, akin to the CMT2D defect (Figure 4, A and B). This indicates that BDNF availability at nerve terminals modulates axonal endosome transport dynamics. In contrast, anti-BDNF did not exacerbate endosome trafficking in *Gars^C201R/+^* mutants (Figure 4, A and B), which is consistent with perturbations in BDNF/TrkB signaling causing CMT2D axonal transport disruption.

To corroborate this, we coinjected wild-type muscles with H_C_T-555 and the selective and peripherally restricted pan-Trk inhibitor, PF-06273340 (47). PF-06273340 binds to the extracellular domain of Trk receptors, restricting their interactions with neurotrophins. Trk inhibition slowed transport in healthy axons of wild-type animals (Figure 4, C and D), validating a role for local Trk signaling in maintaining healthy axonal endosome trafficking.

There are several key signaling pathways downstream of BDNF/TrkB, including AKT, PLCγ1, and ERK1/2 (22). To identify which of these are affected in CMT2D nerves, we extracted sciatic nerves from wild-type and *Gars^C201R/+^* mice that received injections of vehicle or BDNF into the tibialis anterior and gastrocnemius muscles. It should be noted that motor neurons innervating these muscles constitute only a small fraction of the total sciatic nerve; hence, BDNF-induced signaling changes triggered by this experimental procedure are predicted to be small. Probing for total and phosphorylated forms of key proteins in each of these signaling nodes, we identified that under basal conditions, only ERK1/2 activation was impaired in CMT2D nerves (Supplemental Figure 6), suggesting that this pathway contributes to the *Gars^C201R/+^* transport defect. Consistent with this (48), we showed that BDNF treatment enhanced ERK1/2 phosphorylation in both genotypes but to a lesser extent in CMT2D mice. This was again selective, since AKT and PLCγ1 phosphorylation were unchanged.

To test the hypothesis that impaired ERK1/2 activation contributes to compromised endosome transport, we injected the ERK1/2 inhibitor, refametinib (49), into wild-type muscles and analyzed the axonal transport of signaling endosomes. We found that ERK1/2 inhibition caused a stark disruption in trafficking (Figure 4, E and F), validating the importance of ERK1/2 activation for maintaining healthy speeds of signaling endosome axonal transport in motor neurons in vivo.

To identify whether a local, rather than systemic, effect of ERK1/2 inhibition at distal motor neuron terminals causes transport disruption, we coadministered H_C_T-555 with refametinib into hind limb muscles on one side of the body and H_C_T-555 with vehicle control on the other (Supplemental Figure 7A), followed by axonal transport measurement. We found that local ERK1/2 inhibition within the injected muscle perturbed transport but had no impact on endosome trafficking in contralateral sciatic nerves (Supplemental Figure 7, B–E). This result was corroborated by performing similar experiments using IgY control and anti-BDNF antibodies (Supplemental Figure 7, F–J).

These data in wild-type mice indicate that perturbation of BDNF/TrkB signaling via ERK1/2 inhibition at peripheral nerve terminals can disrupt signaling endosome transport in otherwise-healthy motor axons, thus replicating the CMT2D phenotype.

### Applying recombinant BDNF to CMT2D muscles restores in vivo axonal transport.

**Figure 5:**
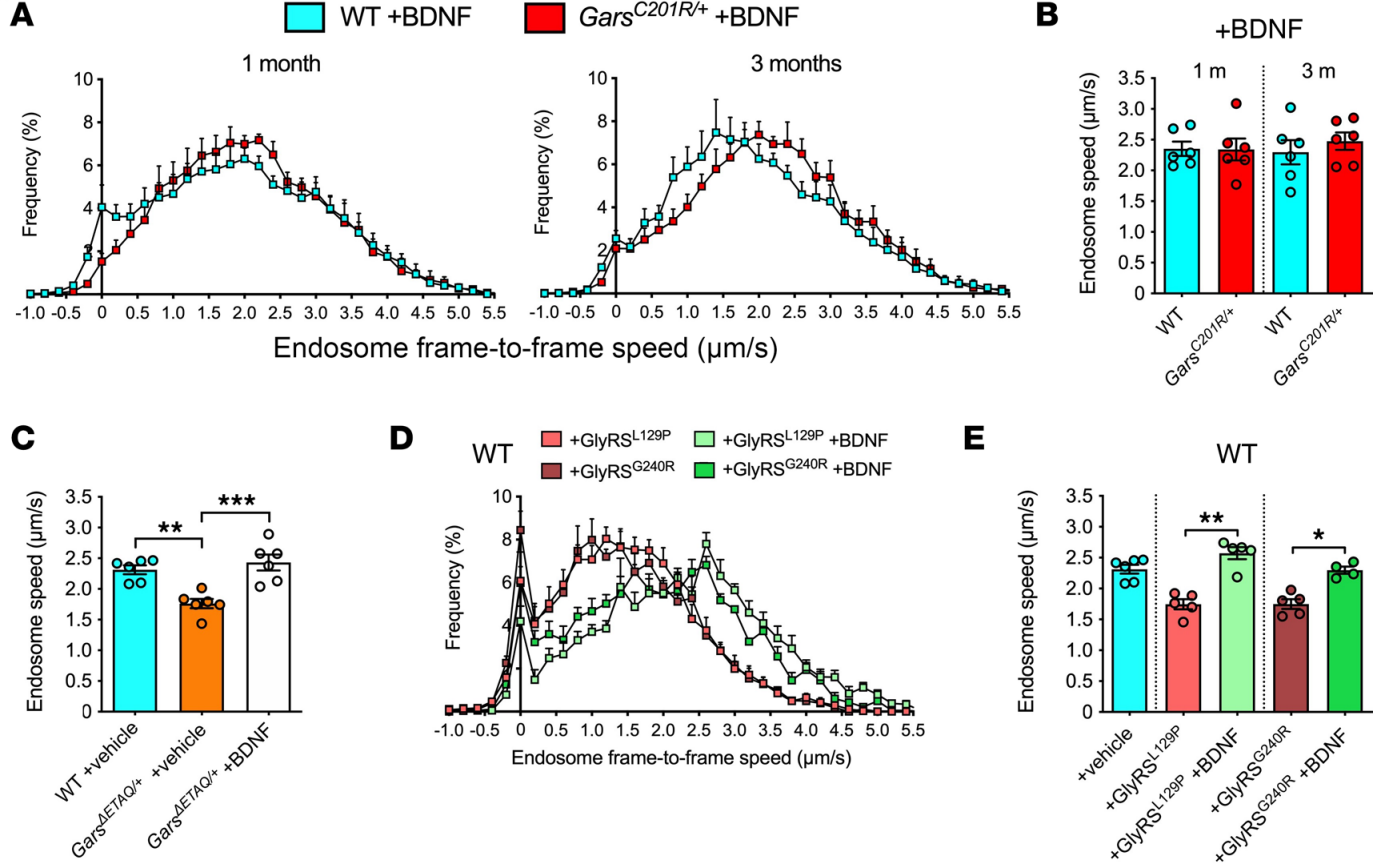
Augmenting BDNF in CMT2D muscles restores in vivo axonal transport. (**A**) Endosome frame-to-frame speed histograms of wild-type and *Gars^C201R/+^* mice aged 1 and 3 months 4–8 hours posttreatment with intramuscular BDNF injections. (**B**) BDNF rescues in vivo axonal transport of signaling endosomes in *Gars^C201R/+^* mice (*P* = 0.875, 1-way ANOVA). (**C**) BDNF corrects transport in 1-month-old *Gars^ΔETAQ/+^* mice (*P* < 0.001, 1-way ANOVA). (**D** and **E**) The axonal transport impairment induced by human CMT2D-causing GlyRS is prevented by exogenous recombinant BDNF (**E**, *P* < 0.001 Kruskal-Wallis test). Mice were aged 1 month. For all graphs, *P < 0.05, **P < 0.01, ***P < 0.001, Šídák’s/Dunn’s multiple comparisons test; *n* = 4–6. See Supplemental Figure 8.

BDNF appears crucial for maintaining axonal dynamics of signaling endosomes generated at peripheral nerve terminals. We thus performed intramuscular injections of 25 ng recombinant mBDNF and reassessed endosome transport in mutant *Gars* mice. In line with the restoration of ERK1/2 activation in CMT2D sciatic nerves (Supplemental Figure 6), BDNF treatment rescued in vivo axonal transport in *Gars^C201R/+^* mice to wild-type levels at 1 and 3 months of age (Figure 5, A and B). Moreover, we showed that injection of BDNF at the late stage of 13–14 months enhanced *Gars^C201R/+^* endosome transport, whereas it had no effect in wild-type mice (Supplemental Figure 8). Also, the trafficking deficit observed in 1-month-old *Gars*^ΔETAQ/+^ mice was fully corrected upon intramuscular injection of BDNF (Figure 5C). We administered BDNF to ascertain whether it could restrict the non–cell-autonomous disruption of endosome transport caused by human mutant GlyRS. Doing so reversed the transport deficit (Figure 5, D and E), suggesting that boosting BDNF levels can overcome the negative, non–cell-autonomous effect on axonal endosome mobility caused by mutant GlyRS at peripheral nerve terminals.

**Figure 6:**
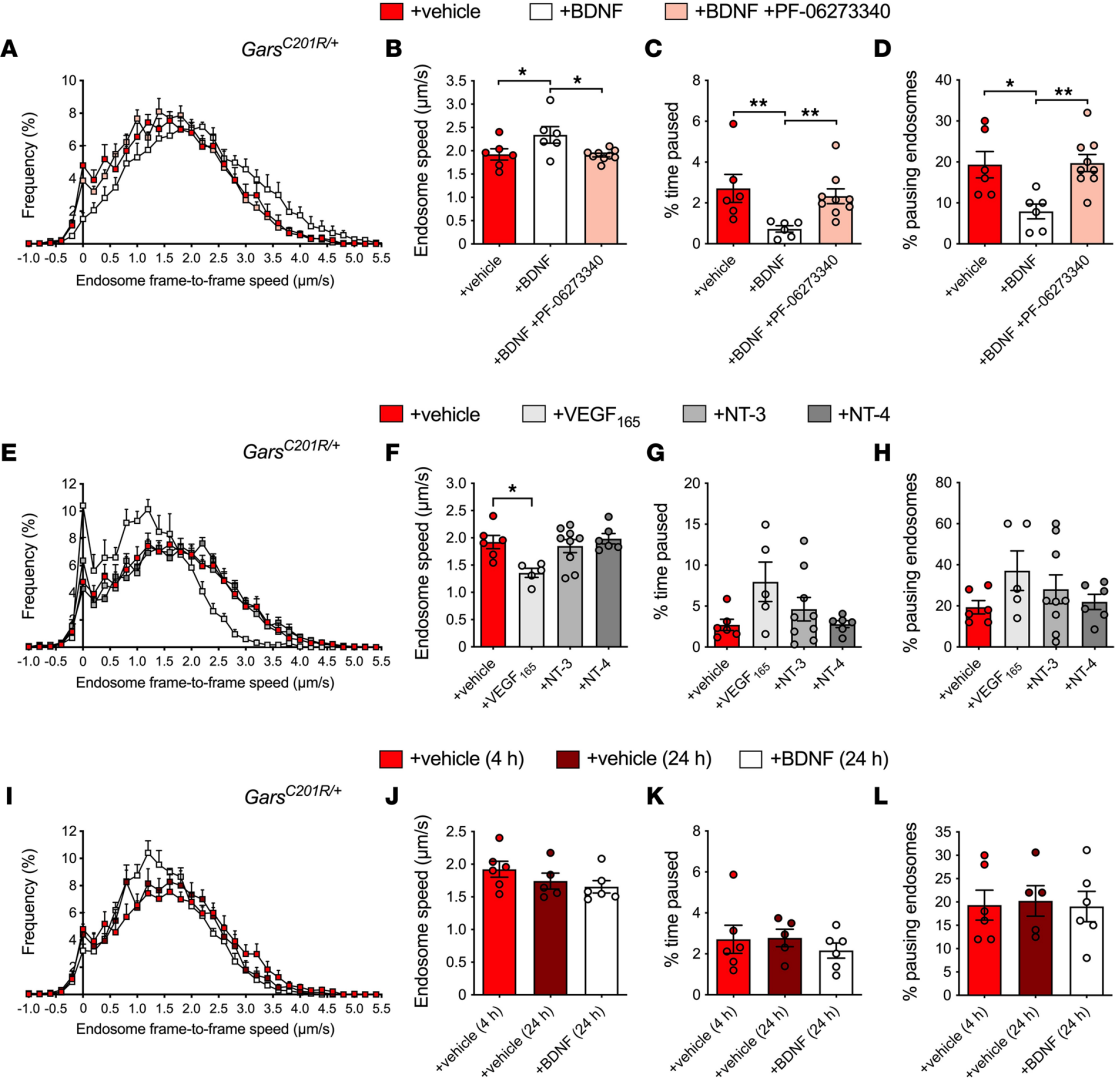
BDNF specifically rescues axonal transport in CMT2D mice via a Trk-dependent mechanism. (**A**) Endosome frame-to-frame speed histograms of 1-month-old *Gars^C201R/+^* mice 4–8 hours posttreatment with vehicle, BDNF, or BDNF plus pan-Trk inhibitor (13 nM PF-06273340). (**B**) Trk inhibition abrogates the rescue effect of BDNF on *Gars^C201R/+^* endosome transport speed (*P* = 0.023). (**C**) Endosomes of *Gars^C201R/+^* mice treated with BDNF and PF-06273340 spend as much time paused as vehicle-treated mice (*P* < 0.001, Kruskal-Wallis test). (**D**) Trk inhibition abrogates the positive effect of BDNF on pausing endosomes (*P* = 0.005). (**E**) Endosome frame-to-frame speed histograms of 1-month-old *Gars^C201R/+^* mice 4–8 hours posttreatment with vehicle, VEGF_165_, NT-3, or NT-4. (**F**–**H**) VEGF_165_ impairs *Gars^C201R/+^* endosome transport speed, while NT-3 and NT-4 have no effect (**F**, *P* = 0.011). No significant changes in pausing (**G**, *P* = 0.086; **H**, *P* = 0.323). (**I**) Endosome frame-to-frame speed histograms of *Gars^C201R/+^* mice treated with vehicle or BDNF for 24 hours, rather than 4–8 hours, before imaging. (**J**–**L**) At 24 hours postinjection, BDNF no longer rescues *Gars^C201R/+^* endosome transport speed (**J**, *P* = 0.248), percentage time paused (**K**, *P* = 0.664), or the percentage of pausing endosomes (**L**, *P* = 0.966). Mice were aged P29–P42 (**I**–**L**). For all graphs, data were compared using 1-way ANOVAs, unless otherwise stated; *P < 0.05, **P < 0.01, Šídák’s/Dunn’s multiple comparisons test; *n* = 5–9. The vehicle treatment data are also presented in Figure 1, C and D, and BDNF treatment data in Figure 5, A and B.

To confirm that the Trk receptors are responsible for the observed amelioration of transport caused by BDNF, *Gars^C201R/+^* mice were treated with a combination of BDNF and pan-Trk inhibitor. Adding PF-06273340 to the intramuscular injection abolished the BDNF-mediated rescue of *Gars^C201R/+^* endosome transport (Figure 6, A–D), demonstrating that BDNF regulates axonal trafficking by canonical Trk signaling.

To further assess selectivity of this rescue, we individually injected several recombinant growth factors into muscles of *Gars^C201R/+^* mice. We chose vascular endothelial growth factor 165 (VEGF_165_) because it is critical to motor neuron survival and signals through neuropilin-1 (50), a binding partner of mutant, but not wild-type, GlyRS (11). We also tested NT-3 and NT-4, which bind to TrkC and TrkB, respectively. We did not evaluate the impact of NGF, because TrkA is not expressed at the NMJ (37), and NGF administration can cause rapid and direct sensitization of nociceptors (51), which would cause unnecessary suffering to the treated mice. Unlike BDNF, injection of NT-3 and NT-4 had no effect on *Gars^C201R/+^* transport, whereas VEGF_165_ exacerbated the CMT2D impairment (Figure 6, E–H), suggesting that the action of BDNF on axonal endosome trafficking in neuropathic mice is specific and does not extend to all neurotrophic factors.

To evaluate the temporal impact of BDNF treatment, we imaged in vivo axonal transport of *Gars^C201R/+^* mutants 24 hours after BDNF injection. We observed no difference from vehicle-injected mice (Figure 6, I–L), indicating that the benefit of injected recombinant BDNF is short-lived.

Together, these data indicate that the rescue of endosome axonal trafficking defects by BDNF is specific and occurs via its canonical Trk receptor.

### Muscle-specific BDNF gene therapy rescues CMT2D axonal transport.

To improve the potential for translation of this discovery, we designed an adeno-associated virus (AAV) to constitutively express BDNF in muscle, thereby avoiding the side effects of systemic BDNF upregulation (52). AAV serotype 8 (AAV8), which displays efficient muscle tropism (53), was combined with the muscle-specific promoter *tMCK* (54). First, an AAV8-tMCK-eGFP control virus was shown to be selectively expressed in skeletal muscles and the heart when injected into the peritoneum of wild-type P2 pups (Supplemental Figure 9, A–C). After verifying muscle selectivity of our virus/promoter combination, we performed unilateral injections of AAV8-tMCK-BDNF into the tibialis anterior and gastrocnemius muscles of P11 wild-type mice at several doses (Supplemental Figure 9, D–G). At all concentrations tested, we observed a similar robust upregulation of proBDNF in injected muscles, as well as the adjacent extensor digitorum longus, indicating that our lowest tested dose of 5.0 × 10^10^ vector genomes (vg) per muscle was sufficient to maximally express the transgene.

**Figure 7:**
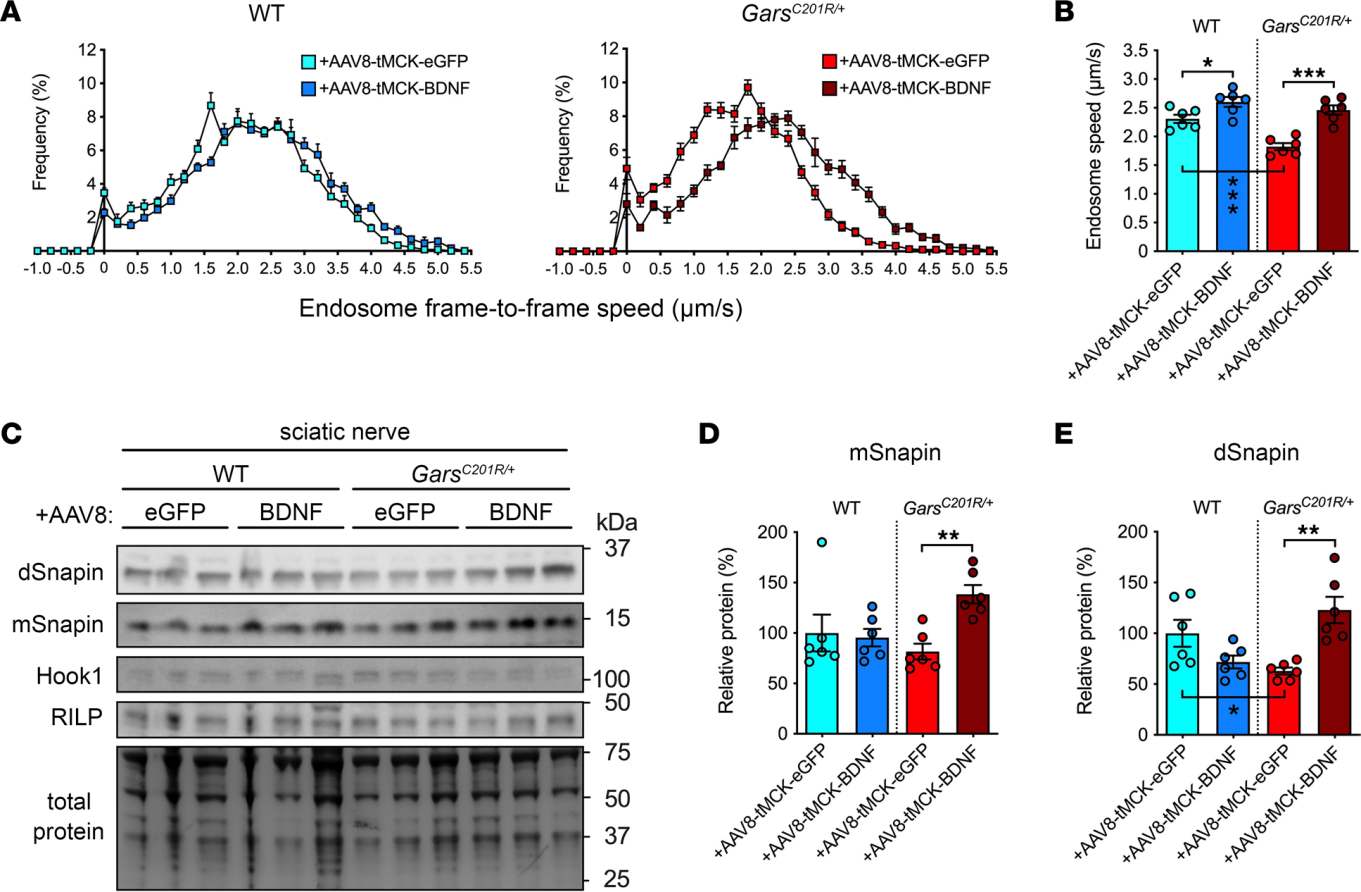
Muscle-specific BDNF gene therapy restores in vivo axonal transport and endosome adaptor levels in CMT2D mice. (**A**) Endosome frame-to-frame speed histograms of P38–P41 wild-type and *Gars^C201R/+^* mice treated at P11 with AAV8-tMCK-EGFP or AAV8-tMCK-BDNF. (**B**) The BDNF gene therapy rescues *Gars^C201R/+^* in vivo axonal transport and increases wild-type endosome speeds (*P* < 0.001). (**C**) Representative Western blot of sciatic nerve lysates from AAV-treated mice probed with antibodies against dynein adaptors. (**D** and **E**) Treatment with AAV8-tMCK-BDNF restores levels of both monomeric (mSnapin; **D**, *P* = 0.016, Kruskal-Wallis test) and dimeric (dSnapin; **E**, *P* = 0.002) Snapin in *Gars^C201R/+^* sciatic nerves but has little effect on Hook1 (*P* = 0.460, not shown) or RILP (*P* = 0.605, not shown). One-way ANOVAs, unless otherwise stated; *P < 0.05, **P < 0.01, ***P < 0.001, Šídák’s/Dunn’s multiple comparisons test; *n* = 6. See Supplemental Figure 9.

Having identified a well-tolerated virus dose that drives observable increases in proBDNF expression, we performed bilateral injections of AAV8-tMCK-BDNF or AAV8-tMCK-eGFP targeting lumbrical/FDB, tibialis anterior, and gastrocnemius muscles of P11 wild-type and *Gars^C201R/+^* mice to analyze the impact on sciatic nerve axonal transport. Successful transgene expression was verified in muscles from all treated animals at the experimental end stage of P38–P41 (Supplemental Figure 9, H–J). Similar to the acute improvement driven by intramuscular injections of recombinant mBDNF, AAV8-tMCK-BDNF rescued axonal transport of endosomes in *Gars^C201R/+^* mice to wild-type levels (Figure 7, A and B). Moreover, the increased BDNF availability also resulted in faster endosome speeds in wild-type mice compared with treatment with AAV8-tMCK-eGFP (Figure 7B). As this increase was not observed in wild-type animals upon intramuscular injection of BDNF (Supplemental Figure 8), this suggests that the amount of BDNF and length of exposure can influence transport in healthy peripheral nerves. Consistent with the increased transport in CMT2D mice, AAV8-tMCK-BDNF also caused a selective increase in availability of the motor adaptor protein Snapin, but not Hook1 or RILP, in mutant sciatic nerves (Figure 7, C–E).

In conclusion, we generated and tested a potentially novel muscle-specific gene therapy for CMT2D that augments proBDNF and mBDNF levels in a tissue-specific manner, resulting in a robust correction of the impaired in vivo axonal transport of signaling endosomes.

## Discussion

Impaired axonal transport has been reported in diverse neurological diseases, yet it is debated whether this is a primary cause of neuropathology or a secondary consequence of a degenerating nervous system (55). The data presented here are consistent with early disruption of signaling endosome transport being one of the contributing factors to CMT2D; however, it remains to be experimentally shown whether this pathomechanism drives disease progression. Through intravital imaging of intact sciatic nerves, we report, for the first time to our knowledge, that axonal transport is impaired in mammalian peripheral neuropathy in vivo. The disturbance manifests by 1 month of age in 2 mouse models of CMT2D, coinciding with degeneration of the NMJ in the most severely affected muscles (31). As the uptake of the retrograde axonal transport probe into axonal endosomes is dependent on the presence of motor nerve terminals, these identified trafficking impairments will underrepresent the severity of disruption, because NMJs in hind limb muscles become progressively denervated in mutant *Gars* mice (31, 33). This feature of our assay also provides an explanation of why the transport deficit does not appear to worsen with age, since at each time point we can assess transport only in motor neurons that have a functional connection with the muscle. Further supporting a role in neuropathy, in vivo endosome trafficking in median and ulnar nerve axons was unaffected in *Gars^C201R/+^* mice, even at a later disease stage, which is a result consistent with the observation that motor function and NMJ integrity are relatively spared in CMT2D mouse forelimbs (33). For now, it remains to be determined whether transport of other cargoes, such as mitochondria, is disrupted in CMT2D mice in vivo. This is a particularly intriguing possibility, given the recent discovery that endosomes serve as platforms for translation to maintain mitochondrial function within axons (56, 57).

**Figure 8:**
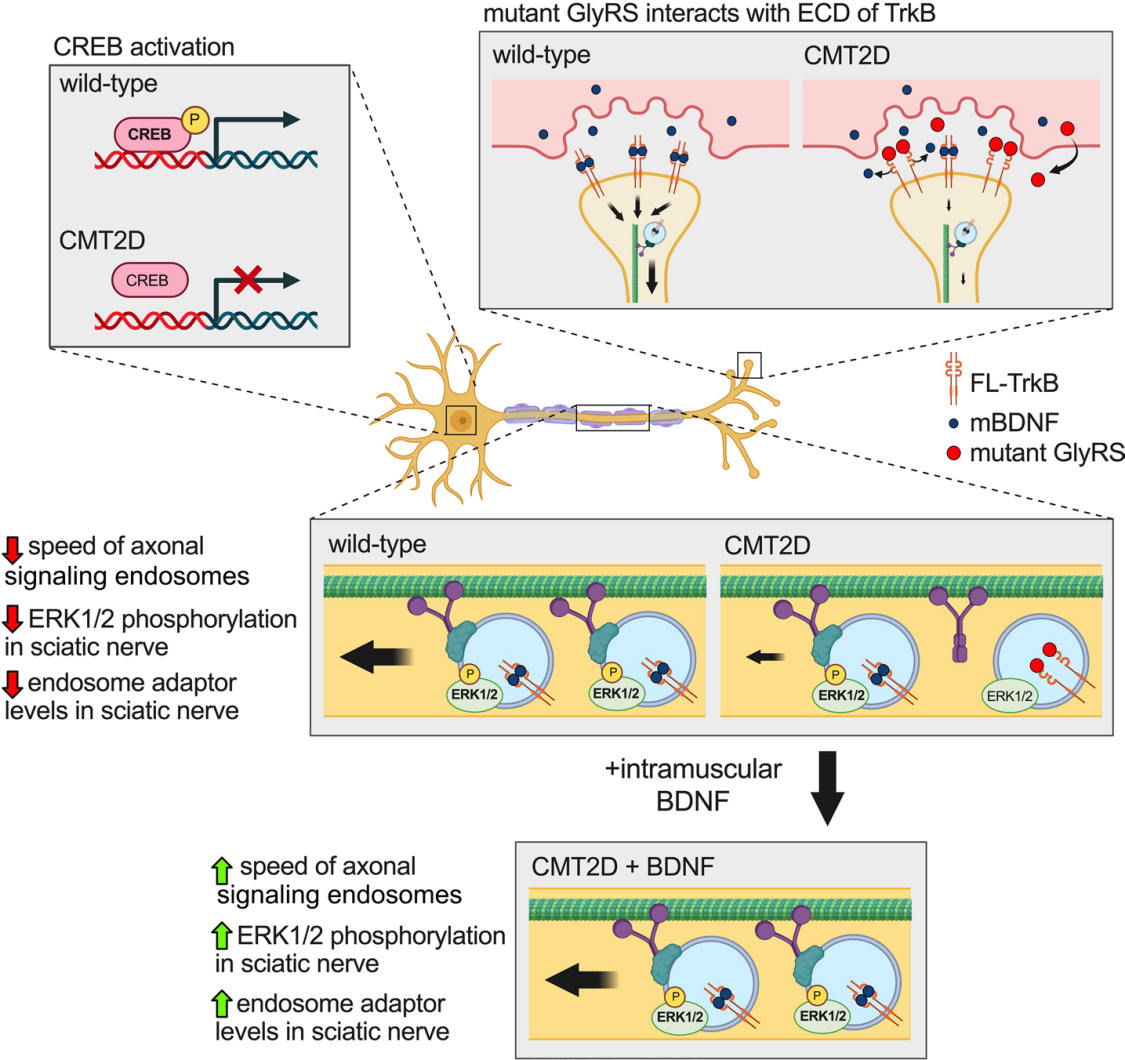
Boosting muscle BDNF rescues impaired axonal transport of signaling endosomes in CMT2D mice. *Top right*: Mutant GlyRS aberrantly interacts with the extracellular domain (ECD) of TrkB, the main neurotrophin receptor found at the NMJ. The availability of FL-TrkB in wild-type muscles correlates with the extent of denervation in CMT2D mice, such that higher FL-TrkB levels are associated with reduced NMJ innervation in neuropathy. *Middle and top left*: CMT2D mice display reduced speed of signaling endosome axonal transport in sciatic, but not median/ulnar, nerves (*middle*), which is associated with reduced ERK1/2 phosphorylation and decreased endosome adaptor levels in sciatic nerves (*middle*), as well as dampened CREB activation (*top left*). *Bottom*: Injection of mBDNF or AAV8-tMCK-BDNF, but not NT-3, NT-4, or VEGF_165_, into CMT2D muscles completely restores physiological axonal transport in vivo. Figure created using https://www.biorender.com.

We hypothesize that interactions between mutant GlyRS and the extracellular domain of TrkB cause the endosome trafficking impairment in motor axons, dampening neurotrophin signaling, which may be contributing to the slowly progressive peripheral nerve degeneration characteristic of CMT (Figure 8). After verifying that GlyRS^ΔETAQ^ aberrantly associates with TrkB, we showed that intramuscular injection of 2 CMT2D-causing mutant, but not wild-type, GlyRS proteins impaired endosome transport in otherwise-healthy motor axons of wild-type mice. This is consistent with mutant GlyRS being the pathological driver at the nerve-muscle interface, altering the regulation of axonal transport of signaling endosomes. Enabling this possibility, GlyRS is secreted from several cell types, including muscles (11, 12, 58, 59), and accumulates at the NMJ before degeneration in a *Drosophila* CMT2D model (12, 15). TrkB is the principal neurotrophin receptor at the mammalian NMJ; thus, it is perhaps no coincidence that its disruption via dominant-negative manipulation (37) or heterozygous knockout (60) disturbs NMJ integrity similar to what is observed in mutant *Gars* mice. Supporting that impaired neurotrophin signaling plays a role in selective peripheral nerve pathology (33), we found a correlation between the availability of FL-TrkB in wild-type muscles and NMJ denervation in CMT2D mice; muscles displaying the greatest NMJ pathology possessed the highest levels of FL-TrkB and the lowest levels of BDNF at the synapse, which would provide ideal conditions for extracellular mutant GlyRS to influence physiological BDNF/TrkB signaling.

We also evaluated potential BDNF/TrkB signaling impairments by assessing activation of the transcription factor CREB in both lumbar and thoracic motor neurons, as well as levels of endosome adaptor proteins in sciatic and median/ulnar nerves. These anatomical studies were aimed to assess whether CMT2D mouse phenotypes are systemic or region specific. We discovered that CREB phosphorylation was selectively disrupted in nuclei of lumbar motor neurons and that levels of the endosome adaptors Snapin and RILP were reduced in sciatic nerves alone. Therefore, NMJ degeneration (33), impaired motor function (33), sensory disruption (13, 21), reduced FL-TrkB availability, dampened CREB signaling, and diminished endosome adaptor levels are all associated with the deficit in signaling endosome trafficking, suggesting that compromised BDNF/TrkB signaling and disturbed axonal transport are integral features of CMT2D pathology.

While impaired protein synthesis in motor neuron cell bodies may be a primary driver of disease (17, 18), the extent of these deficits does not change across spinal levels, dissociating the disruption of protein synthesis from neuropathology. It is therefore possible that aberrant protein interactions at nerve terminals, as well as within axons (14), provide an additional layer of complexity to disease pathogenesis, whereby disturbances in axonal transport drive the selective peripheral degeneration of motor and sensory neurons. In addition, the observation that mutant GlyRS aberrantly associates with HDAC6 to decrease microtubule acetylation (14) suggests that multiple disease mechanisms converge to drive CMT2D pathology.

Acutely increasing the availability of mBDNF in muscle consistently rescued in vivo endosome trafficking in mutant *Gars* axons through its canonical receptor TrkB. Moreover, commensurate with a direct competition between the 2 proteins at axon terminals, mBDNF injected into wild-type muscles was able to overcome the negative impact of mutant GlyRS on endosome transport. Treatment of CMT2D mice with VEGF_165_, NT-3, or NT-4 did not correct the phenotype, indicating that this effect is specific, not a generic feature of factors promoting neuron survival. Supporting the specificity of this rescue, we recently demonstrated that mBDNF is incapable of improving endosome axonal transport defects in SOD1^G93A^ mice modeling amyotrophic lateral sclerosis (61). Crucially, dampening BDNF/TrkB signaling in wild-type muscles, through BDNF sequestration or pan-Trk inhibition, slowed endosome transport in otherwise-healthy neurons. This verified a role for BDNF/TrkB signaling in regulating axonal transport of signaling endosomes.

It is unknown how perturbations in BDNF/TrkB signaling impair axonal endosome speeds in CMT2D; several not mutually exclusive possibilities will be addressed in follow-up studies. For instance, BDNF has been shown to facilitate the recruitment of the intermediate chain of cytoplasmic dynein to signaling endosomes, but not mitochondria, in an ERK1/2 phosphorylation–dependent manner (62). Similarly, increased local translation of cytoplasmic dynein and its adaptors (e.g., Snapin) is driven by peripheral neurotrophin signaling (63, 64), perhaps through redistribution of ribosomes at the axon terminal (65). The blockade of BDNF signaling through TrkB by mutant GlyRS may thus reduce association of the retrograde motor with signaling endosomes, which would be consistent with mutant *Gars* sciatic nerves displaying reduced ERK1/2 phosphorylation and endosome adaptor levels, both of which are rescued by intramuscular administration of BDNF. As multiple dynein motors can interact with and direct the transport of retrograde cargoes for faster movement (66, 67), it is possible that fewer dynein complexes are bound to endosomes in CMT2D axons, causing the slowdown of signaling endosomes.

NT-3 was recently shown to alleviate neuropathology in CMT2D mice when expressed in muscles using AAV1 (68). Similarly, lentivirus-mediated delivery of VEGF_165_ into muscle also partially improves mutant *Gars* motor function (11). These findings, coupled with our data showing that neither growth factor was able to acutely improve axonal transport in CMT2D mice, suggest that restoring axonal endosome trafficking is dispensable for modest improvements in neuropathy and that CMT2D results from impairments in several pathways and processes. The beneficial effect observed with VEGF_165_ and NT-3 in the absence of axonal transport restoration may be due to systemic effects or modulation of non-neuronal components of the motor unit, e.g., muscles or Schwann cells. Alternatively, the lack of transport correction with NT-3, as well as NT-4, may be due to the dose being too low or treatment too short, such that higher levels delivered for prolonged periods may improve endosome trafficking, as observed in this study with BDNF treatment of wild-type mice. NT-3 has previously been shown to bind and activate TrkB during sensory neuron development (69), albeit with a lower affinity than BDNF (70, 71), which would be consistent with this hypothesis; however, it is unknown whether the promiscuity of NT-3 in the motor nervous system is preserved in adulthood. On the other hand, NT-4 has a higher affinity for TrkB than NT-3 and even BDNF (71), suggesting that NT-3 and NT-4 do not regulate endosome axonal transport. This is consistent with the finding that NT-4 binding to TrkB elicits different effects than BDNF (72, 73).

The same lack of response cannot be shown for VEGF_165_, which in our hands exacerbated the CMT2D transport defect. We hypothesize that the further reduction in endosome speed may be due to competition between VEGF_165_ and the alternative neuropilin-1 ligand semaphorin 3A, which facilitates axonal transport in vitro (74); by increasing VEGF_165_ availability, semaphorin 3A may be displaced and no longer able to affect trafficking. Irrespective of the lack of transport improvement, neither NT-3 nor VEGF_165_ treatment results in complete rescue of neuropathy (11, 68), indicating that alternative therapeutic strategies are required to treat this disease and that a combinatorial approach including BDNF may produce the greatest phenotypic improvements.

The restorative effect of BDNF on transport is short-lived, as it was not observed 24 hours postinjection. We therefore developed a gene therapy strategy to constitutively boost mBDNF levels in muscles. This resulted in complete correction of in vivo axonal transport in CMT2D mice up to 1 month posttreatment, which was associated with an increase in levels of the key endosome adaptor protein Snapin. Further work is underway to determine the impact of enhancing the long-term availability of BDNF in muscles as a therapeutic strategy for CMT2D, taking into consideration that genetic depletion and overexpression of BDNF in muscle have been shown to affect fiber type proportions and muscle function (75).

Augmenting BDNF in muscles may be beneficial to trafficking disruption in other CMT subtypes. Mutations in several aminoacyl-tRNA synthetase genes cause CMT (8), several of which are associated with structural relaxation of the encoded synthetase and protein misinteractions (76–78), and share pathomechanistic similarities (17, 79, 80). Additionally, CMT is caused by mutations in genes linked to axonal transport (55), including the critical signaling endosome protein Rab7 (81), and transport disruptions are highly prevalent in neuropathy. Moreover, myelinating Schwann cells provide a major source of trophic support to axons (82) and are lost or damaged in type 1/demyelinating CMT diseases. Thus, boosting the availability of specific growth factors such as BDNF in a spatial and time-dependent manner (Figure 8) may address the therapeutic needs of one of the most widespread forms of human neuropathy.

## Methods

### Animals.

Mice were maintained under a 12-hour light/12-hour dark cycle at constant room temperature (≈21°C) with ad libitum water and food (Teklad global 18% protein rodent diet, Envigo, 2018C). Cages were enriched with nesting material, plastic/cardboard tubes, and wooden chew sticks as standard. *Gars^C201R/+^* (RRID: MGI 3849420) and *Gars*^ΔETAQ/+^ mice (provided by Robert W. Burgess, The Jackson Laboratory, Bar Harbor, Maine, USA) were maintained as heterozygous (male) × wild-type (female) breeding pairs on a C57BL/6J background. *Gars^C201R/+^* mice were genotyped as previously described (30). *Gars*^ΔETAQ/+^ mice were genotyped under standard conditions using forward primer 5′-GGTAGTTTACTTGTAACAGGC-3′ and reverse primer 5′-TTTCCAATCTGGGCAGCAGC-3′ (custom-made by Merck). Both female and male mice were used (Supplemental Table 1) because no sex differences in pathology have been observed. Moreover, axonal signaling endosome dynamics do not differ between female and male wild-type C57BL/6J mice (29). Animals sacrificed for 0.5-, 1-, 3-, and 13- to 14-month time points were 15–16, 28–38, 87–104, and 391–426 days old, respectively (Supplemental Table 1). Additional time points were used and are detailed where appropriate. P1 was defined as the day after a litter was first found.

### In vivo axonal transport imaging.

Live imaging of signaling endosome axonal transport was performed using an atoxic binding fragment of tetanus neurotoxin (H_C_T) (26–28). For imaging transport in sciatic nerves, H_C_T-555 was injected into the right lateral gastrocnemius and tibialis anterior muscles of isoflurane-anesthetized mice (Figure 1, A and B). Alternatively, H_C_T-555 was administered into the left forepaw (targeting lumbrical muscles, among others) for assessment of transport in median and ulnar nerves, as described (46). A 10 μL, 26 gauge Hamilton syringe (MilliporeSigma, 20779) or a pulled, glass micropipette (Drummond Scientific, 5-000-1001-X10) was used for intramuscular injections, whereas micropipettes alone were used for forepaw injections. Muscles of the left leg were also injected in experiments designed to assess the site of action of transport-altering treatments; the side of administration for vehicles and treatments was alternated between mice to limit biases. A total of 5–7 μg of H_C_T-555 in phosphate-buffered saline (PBS) was injected per muscle or forepaw in a volume of ≈1.5–2 μL, before allowing animals to recover from the anesthesia. At 4–8 hours postinjection (unless otherwise stated), nerves were exposed under terminal anesthesia and imaged on an inverted LSM780 laser-scanning microscope (ZEISS) within an environmental chamber prewarmed to 37°C. Images (1,024 × 1,024 pixels, 1% laser power) were acquired every ≈3 seconds using a 63× Plan-Apochromat oil immersion objective lens (ZEISS) at 100× digital zoom. Faster frame rates have been used to determine that endosome fission/fusion events have no clear impact on transport analyses.

### Intramuscular injections.

Different substances were premixed and coadministered with H_C_T-555 into the gastrocnemius and tibialis anterior muscles: 25 ng recombinant human GlyRS (WT/G240R/L129P), 250 ng chicken IgY anti-BDNF (R&D Systems, AF248), 250 ng chicken control IgY (R&D Systems, AB-101-C), 13 or 50 nM PF-06273340 (MilliporeSigma, PZ0254) with 25 ng recombinant human BDNF (Peprotech, 450-02), DMSO (MilliporeSigma, D1435), 50 nM refametinib (Generon, HY-14691), 25 ng recombinant human BDNF, 25 ng recombinant human VEGF_165_ (Peprotech, 100-20), 25 ng recombinant human NT-3 (Peprotech, 450-03), and 25 ng recombinant human NT-4 (Peprotech, 450-04). Amounts are per muscle. Recombinant nontagged GlyRS was purified as previously described (11). Drug concentrations were selected based on reported IC_50_ values: 6/2/1 nM PF-06273340 (TrkA/B/C) (47) and 19/47 nM refametinib (ERK1/ERK2) (49). For Western blot analysis of sciatic nerves, similar injections of vehicle or BDNF were performed (where indicated) without adding H_C_T. Sciatic nerves were dissected 6 hours postinjection.

### In vivo axonal transport analysis.

Confocal.czi files were uploaded to ImageJ (https://imagej.net/ij/index.html) and endosome dynamics manually tracked using the TrackMate plugin (83). Only endosomes that could be tracked for ≥5 consecutive frames were analyzed. Endosomes that paused for ≥10 consecutive frames or moved solely anterogradely were excluded. The tracked fraction of endosomes varied depending on how densely populated with fluorescent organelles the axon was (≈1%–50%); nevertheless, endosomes were selected for assessment as they entered the field of view, without prior observation of their movements. Endosome frame-to-frame speeds are presented in frequency histograms (581 ± 16 movements per animal, *n* = 248 across the study). Endosomes usually take 5–15 frames to traverse the field of view, with rare slower endosomes reaching ≈30 frames. To determine mean endosome speed per animal, individual endosome speeds were averaged (60.1 ± 1.6 endosomes per animal, *n* = 248 across the study). An endosome was determined to have paused if it remained stationary (within < 0.1 μm) for 2 consecutive frames. The “% time paused” is a calculation of the time all tracked endosomes remained stationary. The “% pausing endosomes” defines the proportion of endosomes that paused at least once. A minimum of 10 endosomes from at least 3 individual axons were assessed per animal 5–120 minutes from initiating anesthesia (most videos were recorded within 45 minutes). Thick axons were selected to increase the likelihood of imaging motor, rather than sensory, neurons (29).

### Protein extraction and Western blotting.

Tissues for Western blotting were dissected from PBS-perfused and nonperfused mice. Lumbar level 1 (L1) to L5 DRG and whole-mount muscles were excised as previously outlined (84–88). Proteins were extracted from tissues as described (13), except ground tissue samples were incubated on ice in NP-40 lysis buffer (1% [*w/v*] NP-40, 50 mM NaCl, 50 mM Tris-HCl [pH 8.0]) for 1–2 hours rather than 30 minutes. Several FDB, forelimb lumbrical, and hind limb lumbrical muscles from each side of the body were combined to ensure sufficient protein was extracted. Western blotting was performed following published protocols (13), with primary and secondary antibodies detailed in Supplemental Tables 2 and 3, respectively. A total of 20 μg of protein from DRG, 20–40 μg from muscles, and 40 μg from sciatic nerves was loaded per well. Densitometric analysis was performed as previously described (89), using GAPDH or total protein stained with 0.1% Coomassie Brilliant Blue R-250 (Thermo Fisher Scientific, 20278) as the loading control (90). Hook1 bands at ≈110 kDa and ≈90 kDa were quantified in sciatic and median/ulnar nerves, respectively. Protein phosphorylation levels were calculated relative to the total protein (e.g., p-ERK1/2 relative to ERK1/2). Whole-mount muscle protein levels were correlated with previously published *Gars^C201R/+^* NMJ denervation (*n* = 6–8) (33).

### In vitro pull-down assay.

The recombinant human TrkB-Fc chimera, consisting of the extracellular domain of TrkB (Cys32His430; R&D Systems, 688-TK), and control human IgG1 Fc (110-HG, R&D Systems) were bound to Dynabeads Protein G (Thermo Fisher Scientific, 10003D). Whole-cell lysates from NSC-34 cells (CELLutions Biosystems, CLU140) transfected with plasmids encoding human GlyRS^WT^-V5, GlyRS^ΔETAQ^-V5, or vector control were added to the beads and incubated overnight at 4°C. The beads were washed twice with wash buffer (25 mM Tris-HCl [pH 7.4], 150 mM NaCl, 1 mM EDTA, 1% [*w/v*] NP-40, 5% [*w/v*] glycerol) and treated with sodium dodecyl sulphate loading buffer to elute bound proteins. TrkB-Fc and GlyRS-V5 were analyzed by Western blot using anti–human IgG Fc (horseradish peroxidase, preadsorbed) and anti-V5 antibodies, respectively (Supplemental Table 2).

### Spinal cord dissections and staining.

Spinal cords were dissected from 3-month-old mice transcardially perfused with 4% (*w/v*) methanol-free formaldehyde (Thermo Fisher Scientific, 28908) in PBS. Samples were then postfixed for 24–48 hours at room temperature, before washing with PBS and equilibrating in 30% (*w/v*) sucrose (MilliporeSigma, S7903) in PBS for 24–72 hours at 4°C. Using the lumbar enlargement to guide, the L1 to L5 and thoracic level 4 (T4) to T8 segments of the cord were collected and frozen in Tissue-Tek O.C.T. (Sakura Finetek, 4583). Samples were kept at –80°C, until 30 μm sections were cut with an OTF cryostat (Bright Instruments) and collected onto 5 parallel series of polysine-coated slides (VWR, 631-0107). For staining, sections were permeabilized for 10 minutes using 0.3% (*w/v*) Triton X-100 (MilliporeSigma, T8787) in PBS before blocking for 30 minutes in permeabilization buffer containing 5% (*w/v*) bovine serum albumin (MilliporeSigma, A2153), and probing overnight at 4°C with primary antibodies (Supplemental Table 2) in blocking solution. The following day, slides were washed 3 times with PBS, before incubating with secondary antibodies (Supplemental Table 3). Sections were then washed 3 times with PBS, mounted in Fluoromount-G (Thermo Fisher Scientific, 00-4958-02), and covered with 22 × 50 mm cover glass (VWR, 631-0137). Slides were kept at 4°C to set before imaging.

### Immunofluorescence analysis of spinal cords.

Motor neurons from L3–L5 and T4–T6 spinal cord levels were analyzed. Motor neuron counts were performed by assessing the number of ChAT-positive neurons per ventral horn and calculating an average across 10 slides. Cell body areas were measured from maximum intensity–projected *Z*-stack images by drawing around the circumferences of ChAT staining using the freehand tool. A total of 58.0 ± 2.3 cell bodies were assessed per spinal cord segment. To determine average CREB and p-CREB staining intensities, maximum intensity–projected *Z*-stack images were manually thresholded and smoothened and TDP-43–positive nuclear masks created. These were the used to measure the average fluorescence intensity of CREB (53.8 ± 3.6 nuclei per mouse) and p-CREB (50.0 ± 6.0 nuclei per mouse) in nuclei of ChAT-positive neurons to obtain mean values for each mouse. Sections treated only with secondary antibody were also processed for each animal (17.8 ± 0.8 nuclei per mouse) to generate individual mean values that were subtracted from the mean fluorescence intensities of each mouse to remove background. The resulting values were then used to calculate relative nuclear fluorescence intensities for both CREB and p-CREB separately. To calculate the ratio of p-CREB to CREB, the relative p-CREB value for each mouse was divided by the relative CREB value and multiplied by 100. The areas of motor neuron nuclei were also measured using TDP-43 masks (114.3 ± 6.0 per mouse). TDP-43 was used rather than DAPI, because the fluorescence intensity of the latter is low in motor neurons compared with other cells of the spinal cord, whereas TDP-43 clearly highlights motor nuclei (Supplemental Figure 4C). All sections probed with anti-CREB or anti–p-CREB were processed and analyzed in parallel with fluorescence values calculated relative to wild-type.

### Immunofluorescence analysis of NMJs.

Dissected whole-mount muscles were stained as described in detail elsewhere (85). All primary and secondary antibodies are detailed in Supplemental Tables 2 and 3, respectively. Alexa Fluor 647 α-bungarotoxin (α-BTX, Life Technologies, B35450) was used at 1:1,000 to identify postsynaptic acetylcholine receptors. Relative BDNF levels at the NMJ were measured from maximum intensity–projected *Z*-stack images by drawing around the circumferences of α-BTX staining using the freehand tool. Levels of BDNF fluorescence within the α-BTX mask were assessed using the Integrated Density function in ImageJ. The average background fluorescence was subtracted from mean values per muscle and then expressed as a percentage relative to the ETA muscle. An average of 20 ± 1.2 NMJs per muscle were analyzed across 3 mice. GFP fluorescence was imaged in nonfixed whole-mount muscles of AAV-treated mice.

### Muscle fiber typing.

Tibialis anterior muscles were dissected from PBS-perfused mice and immediately frozen in Tissue-Tek O.C.T. Samples were kept at –80°C, until 30 μm transverse sections were cut with an OTF cryostat and collected onto polysine-coated slides that were then stored at –20°C. Muscle sections were stained with antibodies against MHC isoforms and laminin (Supplemental Tables 2 and 3), as previously described (88). Four sections per muscle were imaged at approximately equal positions throughout the muscle. Fiber types and cross-sectional areas were analyzed using the MyoSight plugin for ImageJ (91). Data were averaged across the 4 sections to get values per animal.

### AAV8-tMCK virus production.

Self-complementary AAV (scAAV) expression plasmids were created by OXGENE. A 745 bp *tMCK* promoter sequence was adapted from those reported by Rodino-Klapac et al. (92) and https://www.addgene.org/105556/ (Supplemental Table 4). The 744 bp coding sequence of human pre-proBDNF was sourced from the European Nucleotide Archive (https://www.ebi.ac.uk/ena/browser/view/AAA69805) (Supplemental Table 4). pSF-scAAV-tMCK-eGFP and pSF-scAAV-tMCK-BDNF plasmids were packaged into AAV8 particles by Charles River Laboratories (previously Vigene Biosciences).

### AAV8-tMCK injections.

Viruses were kept at –80°C in 0.01% (v/v) pluronic F68 surfactant in PBS and diluted in sterile PBS. Viruses were freeze-thawed a maximum of 2 times before injection. For assessing tissue specificity of expression, intraperitoneal AAV8-tMCK-eGFP injections were performed on P2 pups using a Hamilton syringe connected to a 30 gauge needle cannula; 7.0 × 10^10^ vg were injected per mouse in a volume of 5 μL. To identify an appropriate dose, intramuscular AAV8 injections were performed with pulled, glass micropipettes directly through the skin into the gastrocnemius and tibialis anterior of P11 pups under isoflurane-induced anesthesia; 5.0 × 10^10^, 1.0 × 10^11^, or 2.0 × 10^11^ vg were unilaterally injected into each muscle in a volume of 2.5 μL. To evaluate gene therapy efficacy, bilateral AAV injections targeting the hind paw lumbrical/FDB, gastrocnemius, and tibialis anterior muscles of P11 pups were performed under anesthesia; each injection of 2.5 μL contained 5.0 × 10^10^ vg (equating to 3.0 × 10^11^ vg per animal).

### Data availability.

All data are provided in the main text and supplemental materials.

### Statistics.

Data were assumed to be normally distributed unless evidence to the contrary was provided by the Kolmogorov-Smirnov test for normality, while equal variance between groups was assumed. Normally distributed data were statistically analyzed using unpaired and paired *t* tests or 1-way and 2-way ANOVA tests followed by Šídák’s multiple comparisons test. Non-normally distributed data were analyzed using Mann-Whitney *U* tests or Kruskal-Wallis tests followed by Dunn’s multiple comparisons tests. Correlation was assessed using Pearson’s product moment correlation when data were normally distributed or Spearman’s rank correlation if data were nonparametric. Sample sizes, which were predetermined using power calculations and previous experience, are reported in figure legends and represent biological replicates (i.e., individual animals). Means ± SEM are plotted for all graphs. All tests were 2 sided and an α level of *P* < 0.05 was used to determine significance. GraphPad Prism 9 software (version 9.5.1) was used for statistical analyses and figure production.

### Study approval.

Experimentation involving mice was performed under license from the UK Home Office in accordance with the Animals (Scientific Procedures) Act of 1986 and was approved by the UCL Queen Square Institute of Neurology Ethical Review Committee, London, United Kingdom.

## Author contributions

JNS and GS conceived the study. JNS, DVC, SS, TW, YT, RLS, JNSV, ERR, APT, SJW, QZ, and GS performed the experiments. JNS wrote the manuscript and produced the figures, with input from all authors. JNS, XLY, and GS supervised the experiments and acquired the funding.

## Supplementary Material

Supplemental data

## Version 1 03/16/2023

In-Press Preview

## Version 2 05/08/2023

Electronic publication
