# Supplementary material for: Boosting peripheral BDNF rescues impaired in vivo axonal transport in CMT2D mice
Source: JCI Insight. 2023 May 8;8(9):e157191. doi: 10.1172/jci.insight.157191 (PMC10243821; doi:10.1172/jci.insight.157191)
Supplement: Supplemental data [file jciinsight-8-157191-s143.pdf]

Boosting peripheral BDNF rescues impaired in vivo axonal transport in CMT2D mice

Authors: James N. Sleigh[1,2,*], David Villarroel-Campos[1], Sunaina Surana[1,2], Tahmina Wickenden[1†], Yao Tong[3], Rebecca L. Simkin[1], Jose Norberto S. Vargas[1], Elena R. Rhymes[1], Andrew P. Tosolini[1], Steven J. West[4], Qian Zhang[3], Xiang-Lei Yang[3], Giampietro Schiavo[1,2*]

Affiliations:

[1] Department of Neuromuscular Diseases and UCL Queen Square Motor Neuron Disease Centre, UCL Queen Square Institute of Neurology, University College London, London, UK.

[2] UK Dementia Research Institute, University College London, London, UK.

[3] Department of Molecular Medicine, The Scripps Research Institute, La Jolla, California, United States of America.

[4] Sainsbury Wellcome Centre, University College London, London, UK.

Supplemental Figures

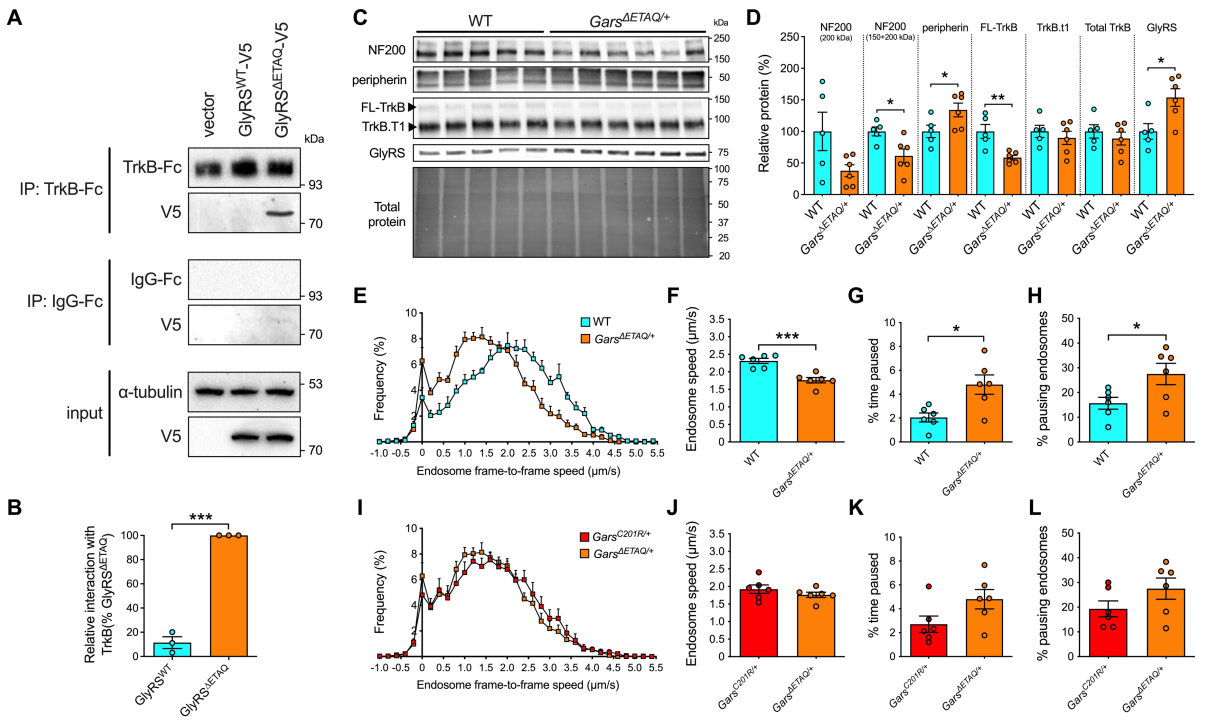

Supplemental Figure 1. ***Gars*$^{\Delta ETAQ/+}$ mice harbouring a CMT2D patient-specific mutation display disturbed axonal transport of signalling endosomes in vivo.** (**A**) Representative western blots from in vitro pull-downs in NSC-34 cells showing that human GlyRS$^{\Delta ETAQ}$, but not GlyRS^{WT}, interacts with the extracellular domain of human TrkB-Fc. *IP*, immunoprecipitation. (**B**) Quantification of the relative interactions between GlyRS^{WT} and GlyRS$^{\Delta ETAQ}$ with TrkB-Fc. n = 3. (**C**) Western blot of L1-L5 DRG lysates from wild-type and *Gars*$^{\Delta ETAQ/+}$ mice aged P82-P143. Samples were probed with antibodies against several proteins that are altered in lumbar DRG of other mutant *Gars* alleles (1, 2). (**D**) Densitometric analyses show that, similar to *Gars*$^{C201R/+}$, levels of total NF200 and full-length TrkB (FL-TrkB) are reduced, and levels of peripherin and GlyRS are increased in *Gars*$^{\Delta ETAQ/+}$ lumbar ganglia. 200 kDa NF200 (P = 0.063), truncated TrkB (TrkB.t1, P = 0.493) and total TrkB (P = 0.462) remain unaltered. n = 5-6. (**E-H**) 1 month-old *Gars*$^{\Delta ETAQ/+}$ mice display slower endosome transport (E and F) and increased pausing (G and H) compared to wild-type animals. (**I-L**) At 1 month, *Gars*$^{\Delta ETAQ/+}$ mice display similar endosome speeds (I and J, P = 0.289),

percentage time paused (K, $P = 0.077$) and percentage pausing endosomes (L, $P = 0.159$) to *Gars*$^{C201R/+}$ mice. $n = 5$-6. For all graphs, $^{*}P < 0.05$, $^{**}P < 0.01$, $^{***}P < 0.001$ unpaired *t*-test; means ± standard error of the mean (SEM) plotted. N.b., wild-type and *Gars*$^{C201R/+}$ transport data are also presented in Figure 1, C and D.

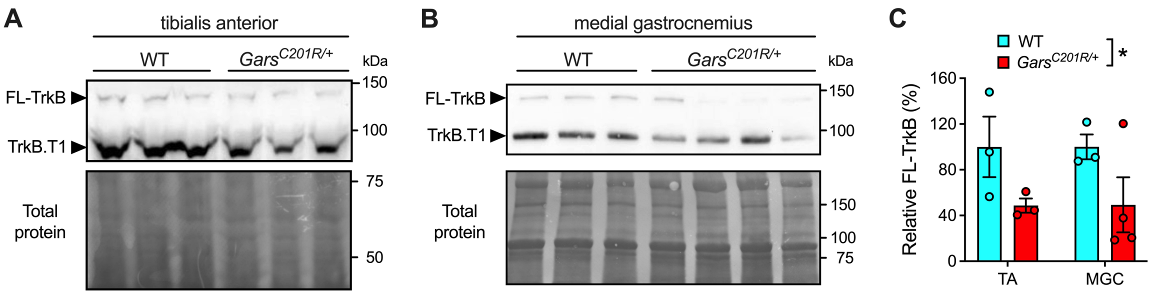

Supplemental Figure 2. FL-TrkB levels are reduced in *Gars*$^{C201R/+}$ muscles at 3 months. (**A** and **B**) Western blots of tibialis anterior (TA, A) and medial gastrocnemius (MGC, B) lysates from 3 month-old wild-type and *Gars*$^{C201R/+}$ mice probed for TrkB. (**C**) Densitometric analyses show that FL-TrkB levels are reduced in *Gars*$^{C201R/+}$ muscles (genotype $^{*}P = 0.035$, muscle $P = 0.988$, interaction $P = 0.988$ two-way ANOVA). $n = 3\text{-}4$. Means ± SEM plotted.

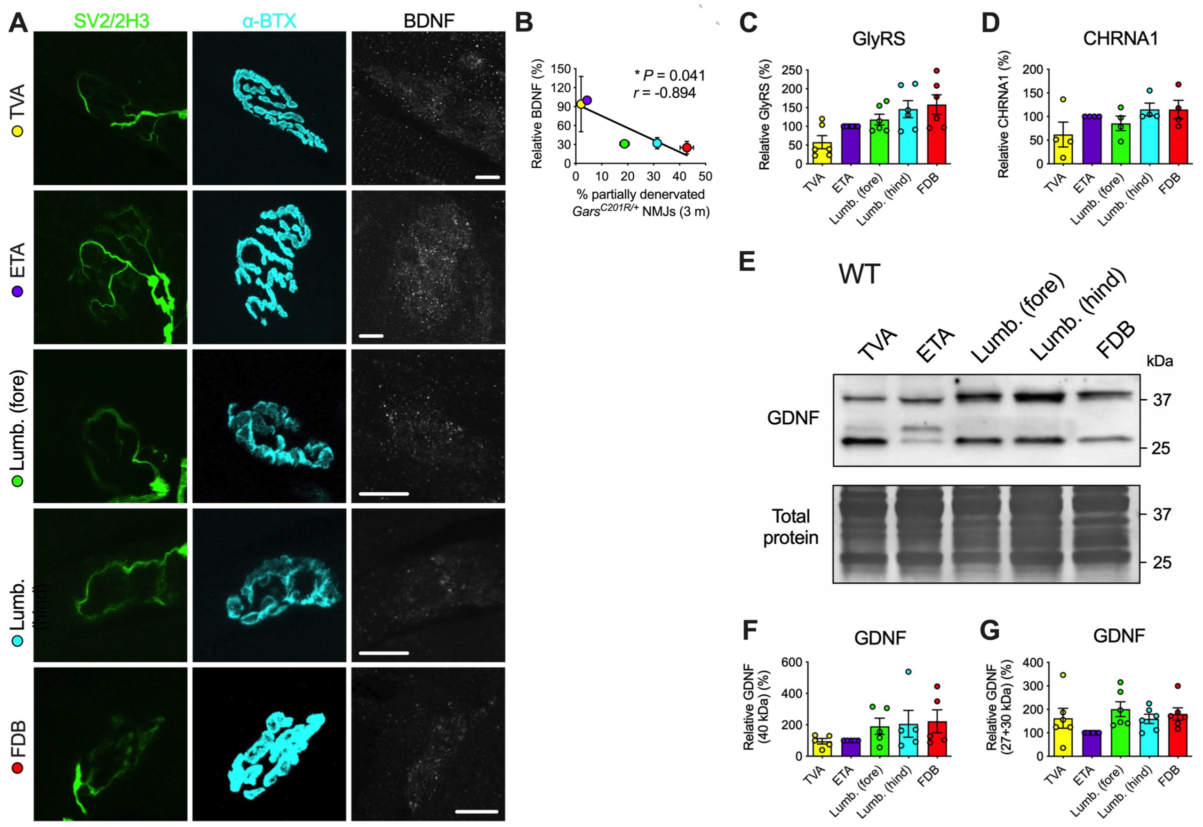

Supplemental Figure 3. Expression of BDNF at the NMJ correlates with CMT2D denervation. (**A**) Representative collapsed z-stack confocal images confirming localization of BDNF (white) at NMJs across wild-type muscles. Terminals of lower motor neurons are visualised using a combination of anti-SV2/2H3 (green) antibodies and post-synaptic acetylcholine receptors with fluorescently-labelled α-bungarotoxin (α-BTX, cyan). Scale bars = 10 µm. (**B**) Relative BDNF expression at wild-type NMJs correlates with the extent of partial denervation previously identified at *Gars*$^{C201R/+}$ NMJs at 3 months (3). Correlation was assessed using Pearson's product moment correlation. *n* = 3. (**C** and **D**) The levels of GlyRS (*P* = 0.101) and CHRNA1 (*P* = 0.239) do not differ between the five wild-type wholemount muscles that display differential vulnerability to NMJ denervation in *Gars*$^{C201R/+}$ mice (3). Representative western blots are presented in Figure 2D. *n* = 4-6. (**E-G**) Representative western blot of wholemount muscle lysates probed for GDNF. Neither 40 kDa GDNF (G, *P* = 0.415), nor 27+30 kDa GDNF (H, *P* = 0.151 one-way ANOVA) differ between muscles. Protein levels

across muscles were compared using Kruskal-Wallis tests, unless otherwise stated. The colour-coding of muscles from panel A is maintained throughout the figure. *n* = 5-6. Wholemount muscles were dissected from wild-type mice aged P132-P140 (A and B) and P77-P83 (C-G).

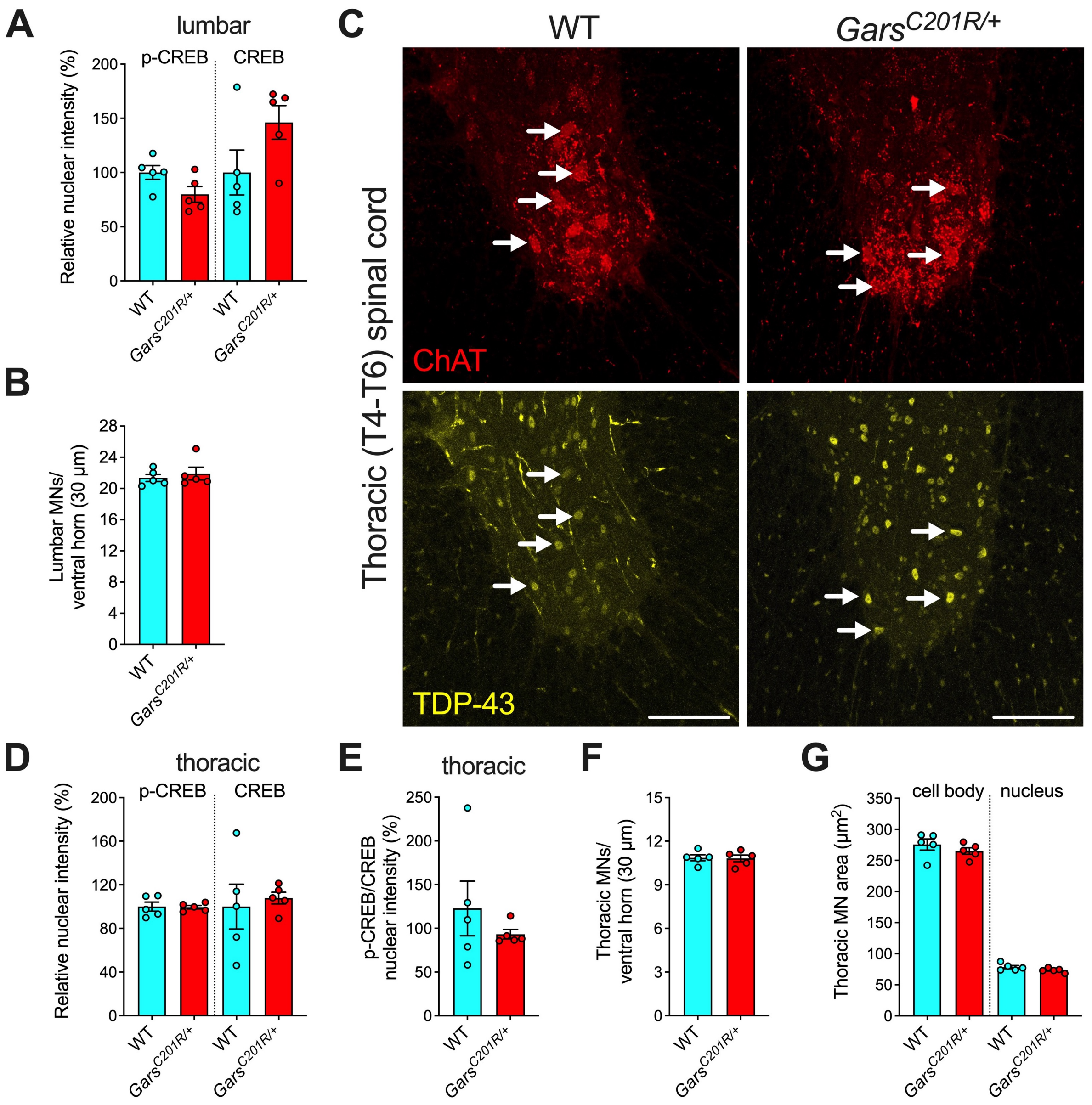

Supplemental Figure 4. Size and CREB signalling are unaffected in *Gars*$^{C201R/+}$ thoracic motor neurons. (**A** and **B**) Nuclear p-CREB (P = 0.071) and CREB (P = 0.112) levels do not significantly differ between 3 month-old wild-type and *Gars*$^{C201R/+}$ lumbar motor neurons (A), and there is no difference in lumbar motor neuron numbers (B, P = 0.730 Mann-Whitney U test). Associated data are presented in Figure 2, E-H. (**C**) Representative collapsed z-stack confocal images of thoracic spinal cord ventral horns from 3 month-old wild-type and *Gars*$^{C201R/+}$ mice. Motor neurons are visualized using ChAT (red) and nuclei using TDP-43

(yellow). Arrows highlight motor neuron nuclei. Scale bars = 100 µm. (**D** and **E**) Thoracic motor neurons show no difference in nuclear p-CREB ($P = 0.908$) or CREB ($P = 0.722$) levels between genotypes (D), and neither does p-CREB activation (E, $P = 0.841$ Mann-Whitney *U* test). (**F**) *Gars*$^{C201R/+}$ mice display no loss of thoracic motor neurons ($P = 0.901$). (**G**) There is no difference in motor neuron cell body ($P = 0.356$) or nucleus ($P = 0.125$) areas between wild-type and *Gars*$^{C201R/+}$ thoracic motor neurons. Lumbar and thoracic spinal cords were dissected and analysed from the same mice. For all graphs, genotypes were compared using unpaired *t*-tests, unless otherwise stated; $n = 5$; means ± SEM plotted.

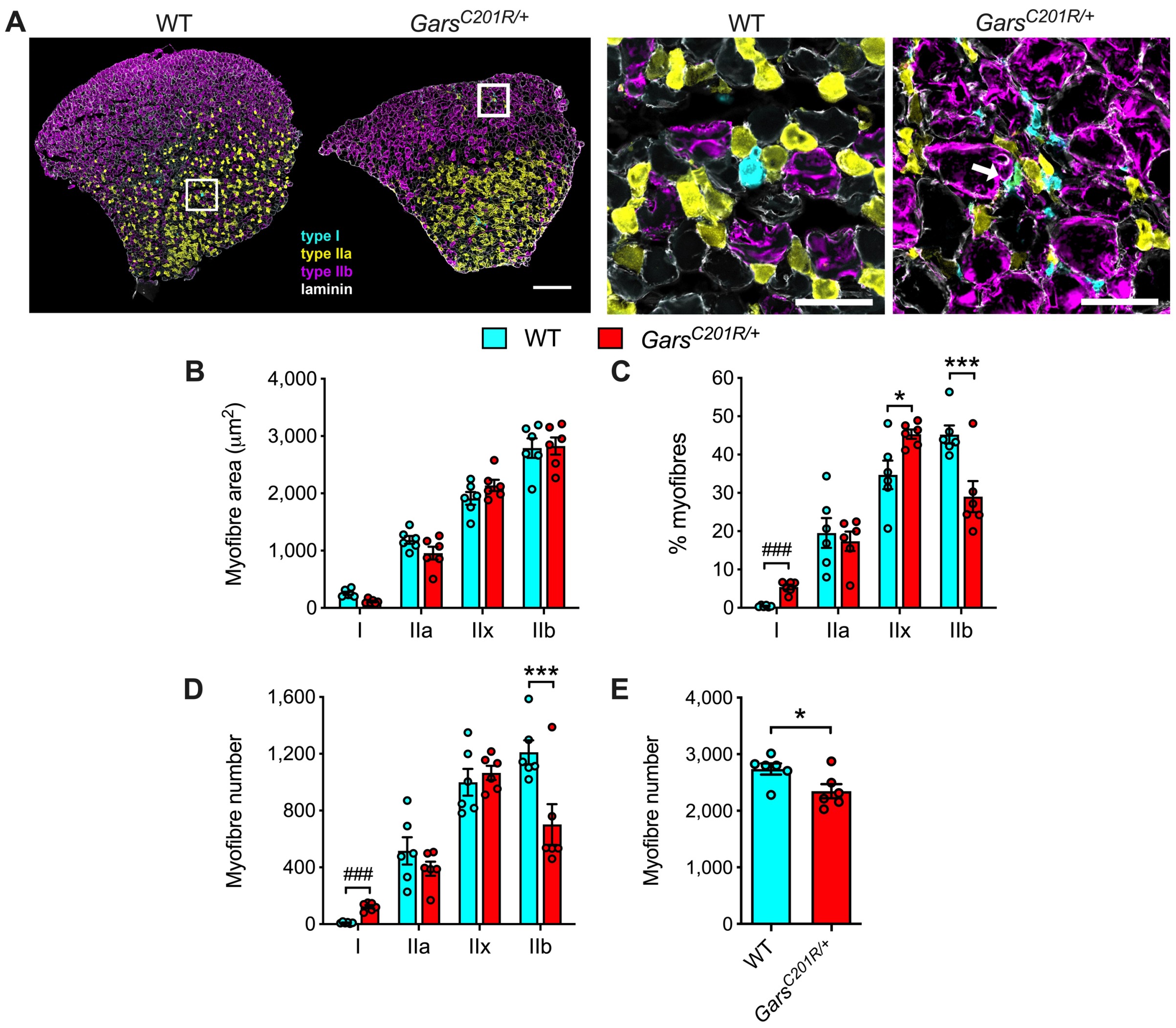

Supplemental Figure 5. Tibialis anterior muscles of *Gars*$^{C201R/+}$ mice possess fewer type IIb myofibres at 3 months. (**A**) Representative single plane, tiled confocal images of tibialis anterior muscle sections from 3 month-old wild-type and *Gars*$^{C201R/+}$ mice stained for myosin heavy chain (MHC) type I (cyan), type IIa (yellow) and type IIb (magenta), as well as laminin (white) to distinguish fibre borders. Unlabelled fibres correspond to MHC type IIx. The two right panels depict the highlighted regions from the left panel. Very few type I fibres were observed in wild-type muscles. *Gars*$^{C201R/+}$ mice displayed fibres positive for both MHC type I and type IIa (I/IIa, white arrow, 2.89 ± 0.63%), which were rarer in wild-type mice (0.11 ± 0.04%; P = 0.007 unpaired t-test). Scale bars = 500 µm (left panel) and 100 µm (right panels). (**B**) There was no difference between genotypes in the cross-sectional area of each subtype of

myofiber (genotype $P = 0.718$, fibre type $P < 0.001$, interaction $P = 0.166$ two-way ANOVA). (**C**) Tibialis anterior muscles from *Gars*$^{C201R/+}$ mice exhibit a lower percentage of type IIb fibres and a higher percentage of type I and type IIx fibres (genotype $P = 0.721$, fibre type $P < 0.001$, interaction $P < 0.001$ two-way ANOVA). $^{*}P < 0.05$, $^{***}P < 0.001$ Šídák's multiple comparison test. $^{\#\#\#}P < 0.001$ unpaired *t*-test. (**D**) *Gars*$^{C201R/+}$ muscles possess fewer type IIb fibres than wild-type (genotype $P = 0.050$, fibre type $P < 0.001$, interaction $P = 0.001$ two-way ANOVA). $^{***}P < 0.001$ Šídák's multiple comparison test. $^{\#\#\#}P < 0.001$ unpaired *t*-test. (**E**) *Gars*$^{C201R/+}$ mice exhibit fewer total fibres in tibialis anterior muscle. $^{*}P < 0.05$ unpaired *t*-test. For all graphs, $n = 6$; means ± SEM plotted.

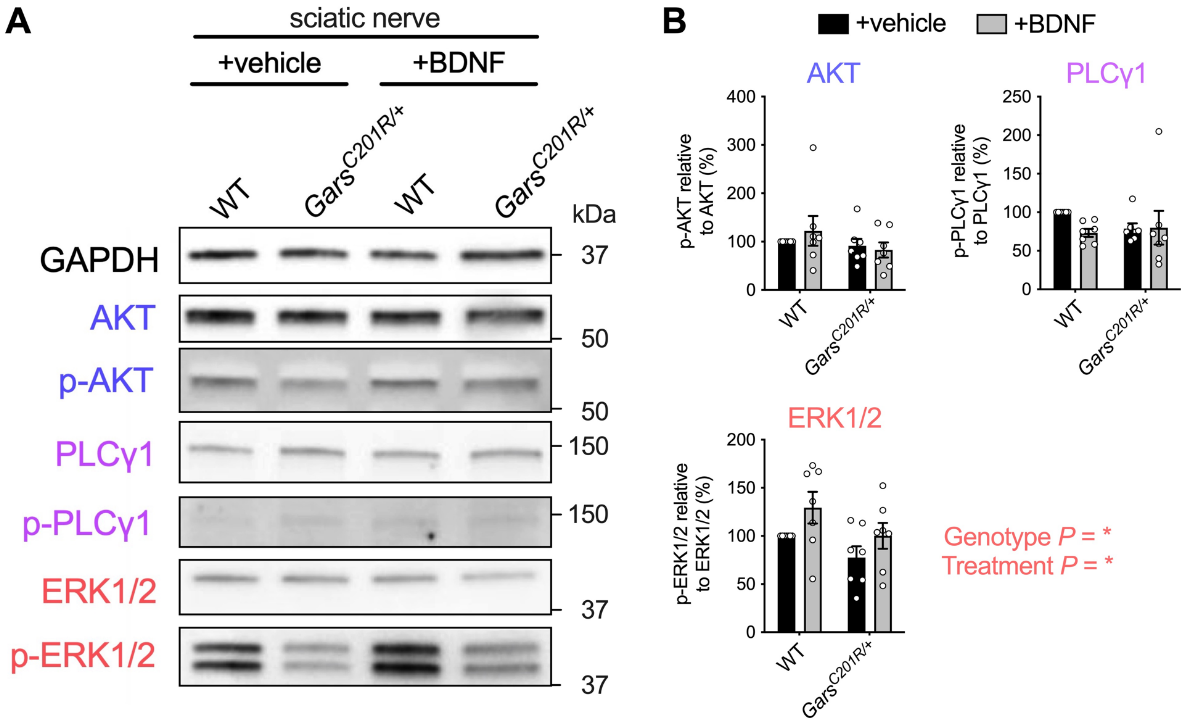

Supplemental Figure 6. Reduced ERK1/2 phosphorylation in *Gars*$^{C201R/+}$ sciatic nerves is restored by intramuscular BDNF. (**A**) Representative western blot of sciatic nerve lysates from vehicle- and BDNF-treated wild-type and *Gars*$^{C201R/+}$ mice probed for total and phosphorylated forms of AKT, PLCγ1 and ERK1/2 – three key signalling nodes downstream of BDNF-TrkB (4). (**B**) Activated ERK1/2 was reduced in vehicle-treated *Gars*$^{C201R/+}$ mice and BDNF increased phosphorylation (genotype $*P = 0.044$, treatment $*P = 0.043$, interaction $P = 0.780$). In contrast, there were no significant differences in phosphorylation of AKT (genotype $P = 0.213$, treatment $P = 0.717$, interaction $P = 0.420$) or PLCγ1 (genotype $P = 0.538$, treatment $P = 0.287$, interaction $P = 0.247$) between genotypes or in response to BDNF. Animals were 1 month-old and 25 ng BDNF was injected per muscle. For all graphs, data were analysed using two-way ANOVAs; $n = 7$; means ± SEM plotted.

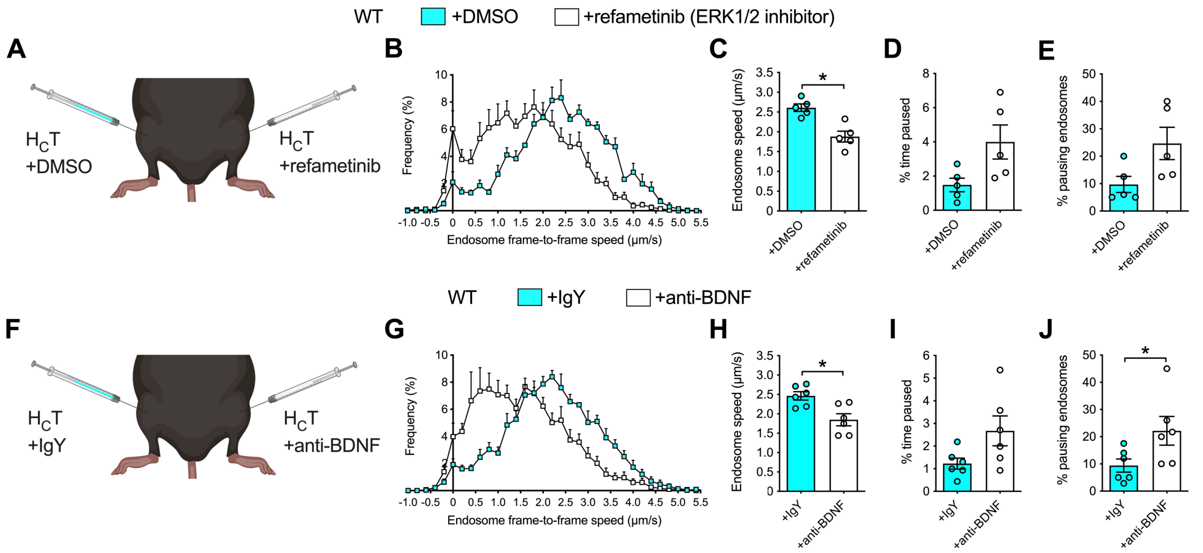

Supplemental Figure 7. ERK1/2 inhibition and BDNF sequestration act locally, not systemically, to impair axonal transport of signalling endosomes. (**A**) Schematic depicting the dual-injection paradigm used in experiments designed to assess whether refametinib acts locally within the injected muscle or systemically, thus impacting transport in the contralateral sciatic nerve. Injections were performed 4-8 h prior to imaging endosome transport. (**B**) Endosome frame-to-frame speed histograms of wild-type mice treated intramuscularly with vehicle (DMSO) into one side of the body and 50 nM refametinib into the other. (**C**) Endosomes only slowed in axons with terminals exposed to refametinib (*P = 0.0254), indicating that ERK1/2 is inhibited only locally. (**D** and **E**) Refametinib does not significantly affect endosome pausing (D, P = 0.098; E, P = 0.131). (**F**) Schematic depicting the dual-injection paradigm used to assess whether anti-BDNF acts locally or systemically. Injections were performed 4-8 h prior to imaging endosome transport. (**G**) Endosome frame-to-frame speed histograms of wild-type mice treated with intramuscular injections of 250 ng IgY or anti-BDNF. (**H-J**) Anti-BDNF antibodies impair transport only in axons with terminals innervating the injected muscles (H, *P = 0.023; I, P = 0.073; J, *P = 0.046). For all graphs, data were compared using paired t-tests; n = 5-6 ; means ± SEM plotted. Animals were P48-P54 (A-E) and 1 month-old (F-J).

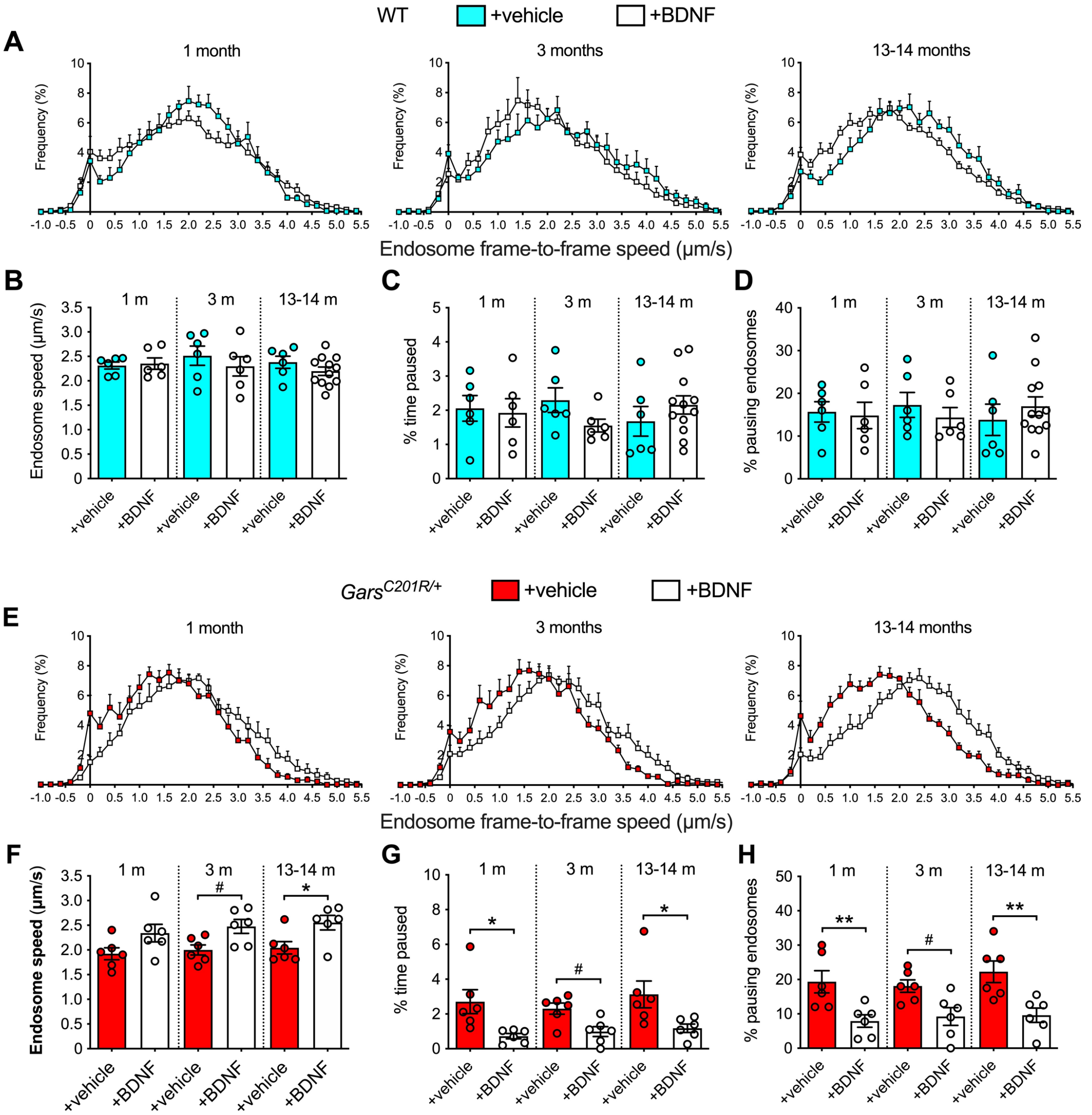

Supplemental Figure 8. BDNF injections correct *Gars*$^{C201R/+}$ transport defects throughout an extended disease course. (**A**) Endosome frame-to-frame speed histograms of wild-type mice aged 1, 3 and 13-14 months treated with intramuscular injections of vehicle or recombinant mature BDNF. (**B-D**) BDNF has no impact on wild-type endosome speeds (B, treatment P = 0.285, age P = 0.685, interaction P = 0.610), percentage time paused (C, treatment P = 0.649, age P = 0.975, interaction P = 0.221) or the percentage of pausing endosomes (D, treatment P = 0.931, age P = 0.980, interaction P = 0.535). (**E**) Endosome

frame-to-frame speed histograms of *Gars*$^{C201R/+}$ mice aged 1, 3 and 13-14 months treated with intramuscular injections of vehicle or BDNF. (**F**) BDNF increases endosome transport speeds in *Gars*$^{C201R/+}$ mice (treatment $P < 0.001$, age $P = 0.484$, interaction $P = 0.945$). (**G**) BDNF reduced the percentage of time that *Gars*$^{C201R/+}$ endosomes pause for (treatment $P < 0.001$, age $P = 0.516$, interaction $P = 0.735$). (**H**) Fewer *Gars*$^{C201R/+}$ endosomes paused upon BDNF treatment (treatment $P < 0.001$, age $P = 0.579$, interaction $P = 0.745$). For all graphs, data were compared using two-way ANOVAs; *$P < 0.05$, **$P < 0.01$ Šídák's multiple comparisons test; $^{\#}P < 0.05$ unpaired *t*-test; $n = 6$-12; means ± SEM plotted. N.b., the vehicle treatment data are also presented in Figure 1, C and D, and BDNF treatment data in Figure 5, A and B.

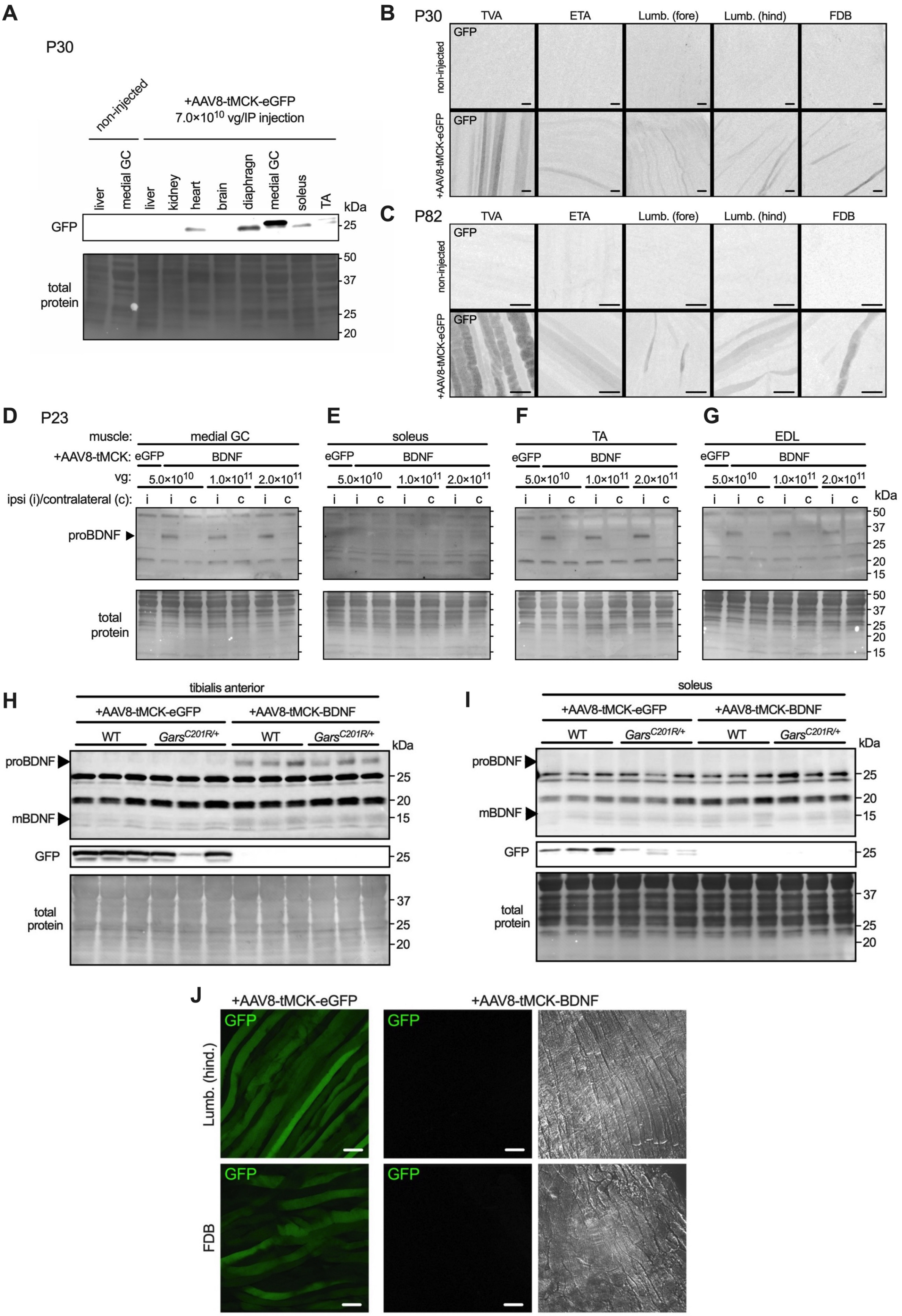
A
P30
non-injected
+AAV8-tMCK-eGFP
7.0×10^10 vg/IP injection
liver
medial GC
liver
kidney
heart
brain
diaphragn
medial GC
soleus
TA
kDa
GFP
total protein
25
50
37
25
20
B
P30
TVA
ETA
Lumb. (fore)
Lumb. (hind)
FDB
non-injected
+AAV8-tMCK-eGFP
GFP
C
P82
TVA
ETA
Lumb. (fore)
Lumb. (hind)
FDB
non-injected
+AAV8-tMCK-eGFP
GFP
D
P23
muscle:
medial GC
+AAV8-tMCK:
eGFP
BDNF
vg:
5.0×10^10
1.0×10^11
2.0×10^11
ipsi (i)/contralateral (c):
i i c i c i c
proBDNF
total protein
E
soleus
F
TA
G
EDL
kDa
50
37
25
20
15
H
tibialis anterior
+AAV8-tMCK-eGFP
+AAV8-tMCK-BDNF
WT
Gars^C201R/+
proBDNF
mBDNF
GFP
total protein
kDa
25
20
15
37
I
soleus
+AAV8-tMCK-eGFP
+AAV8-tMCK-BDNF
WT
Gars^C201R/+
proBDNF
mBDNF
GFP
total protein
kDa
J
+AAV8-tMCK-eGFP
+AAV8-tMCK-BDNF
GFP
Lumb. (hind.)
FDB

Supplemental Figure 9. AAV8-tMCK drives muscle-specific transgene expression. (**A**) Representative western blot showing selective GFP expression in muscles at P30 from a mouse intraperitoneally injected at P2 with AAV8-tMCK-eGFP. Tissues from a non-injected mouse are provided as controls. These findings were replicated in triplicate. (**B** and **C**) Representative single plane confocal images confirming GFP expression in wholemount muscle fibres at P30 (B) and P82 (C) after intraperitoneal AAV8-tMCK-eGFP injection at P2. Scale bars = 100 µm. These findings were replicated in triplicate. (**D**-**G**) Injection of several different concentrations (5.0×10^{10} to 2.0×10^{11} vg/muscle) of AAV8-tMCK-BDNF into the gastrocnemius and tibialis anterior muscles of mice aged P11 resulted in a clear increase in proBDNF in the injected muscles (ipsilateral) by P23 (D and F). No obvious increase in BDNF was observed in contralateral muscles or in the gastrocnemius-adjacent soleus muscle (E). However, the tibialis anterior-adjacent extensor digitorum longus muscle did show increased BDNF (G). *EDL,* extensor digitorum longus; *GC*, gastrocnemius; *IP*, intraperitoneal; *TA*, tibialis anterior; *vg*, vector genomes. These findings were replicated in triplicate. (**H** and **I**) Representative western blots of tibialis anterior (H) and soleus (I) muscles from gene therapy-treated mice probed with anti-GFP and anti-BDNF. Mice treated with AAV8-tMCK-BDNF show a clear increase in proBDNF and mBDNF in tibialis anterior muscles compared with control-treated mice, which show successful GFP expression. A similar pattern is observed in the non-injected soleus, albeit to a lesser extent. $n = 3$ shown; $n = 6$ performed. (**J**) Representative single plane confocal images showing GFP expression in hindlimb lumbrical and FDB muscles of wild-type mice injected with either AAV8-tMCK-eGFP or AAV8-tMCK-BDNF. GFP immunofluorescence was confirmed in all wild-type and *Gars*$^{C201R/+}$ mice injected with control gene therapy viruses. Scale bars = 50 µm.

Supplemental Tables

Supplemental Table 1. Experimental sample sizes and details of the sex and age of all animals used in this study. *AAV*, adeno-associated virus; *d*, days; *BDNF*, brain-derived neurotropic factor; *CMT2D*, Charcot-Marie-Tooth disease type 2D; *DMSO*, dimethyl sulfoxide; *DRG*, dorsal root ganglion; *F*, female; *GFP*, green fluorescent protein; *GlyRS*, glycyl-tRNA synthetase; *h*, hours; *IgY*, immunoglobulin Y ; *M*, male; *MGC*, medial gastrocnemius; *Mut*, mutant; *n*, sample size; *n.a.*, not applicable; *NMJ*, neuromuscular junction; *NT3/4*; neurotrophin 3/4; *TA*, tibialis anterior; $VEGF_{165}$, vascular endothelial growth factor 165; *WT*, wild-type.

Figure	Data	*n*	Sex	Age (d)
1C-D	in vivo transport	6	WT 5M1F / Mut 1M5F	15-16
			WT 3M3F / Mut 3M3F	33-35
			WT 3M3F / Mut 3M3F	90-94
1E-H	WT in vivo transport (+GlyRS)	5-6	+vehicle 3M3F / +GlyRS 5M0F / +L129P 5M0F / +G240R 5M0F	33-37
2A-D	WT muscle western blots	6	0M6F	77-83
2E-H	lumbar spinal cord motor neurons	5	WT 4M1F / Mut 0M5F	104
3A	sciatic nerve western blots	5	WT 4M1F / Mut 4M1F	31-33
3B	median/ulnar nerve western blots	5	WT 3M2F / Mut 3M2F	31-34
3C-F	in vivo transport (median/ulnar)	4-5	WT 2M3F / Mut 1M3F	94-96
4A-B	in vivo transport (+anti-BDNF)	5-6	WT +IgY 2M3F / Mut +IgY 3M2F / WT +anti-BDNF 4M2F / Mut +anti-BDNF 3M3F	30-35
4C-D	WT in vivo transport (+PF-06273340)	5-6	+DMSO 4M1F / +PF-06273340 2M4F	29-35

4E-F	WT in vivo transport (+refametinib)	3	+DMSO 0M3F / +refametinib 0M3F	40-45
5A-B	in vivo transport (+BDNF)	6	WT 3M3F / Mut 3M3F	30-35
			WT 3M3F / Mut 3M3F	90-97
5C	*Gars*$^{\Delta ETAQ/+}$ in vivo transport (+BDNF)	6	WT +vehicle 3M3F / Mut +vehicle 5M1F / Mut +BDNF 3M3F	30-38
5D-E	WT in vivo transport (+GlyRS +BDNF)	4-6	+vehicle 3M3F / +L129P 5M0F / +L129P+BDNF 5M0F / +G240R 5M0F / +G240R+BDNF 4M0F	33-37
6A-D	*Gars*$^{C201R/+}$ in vivo transport (BDNF + PF-06273340)	6-9	+vehicle 3M3F / +BDNF 3M3F / +BDNF+PF-06273340 5M4F	30-35
6E-H	*Gars*$^{C201R/+}$ in vivo transport (VEGF$_{165}$/NT-3/NT-4)	5-9	+vehicle 3M3F / +VEGF$_{165}$ 1M4F / +NT-3 4M5F / +NT-4 3M3F	28-35
6I-L	*Gars*$^{C201R/+}$ in vivo transport (24 h)	5-6	+vehicle(4 h) 3M3F / +vehicle(24 h) 2M3F / +BDNF(24 h) 4M/2F	29-42
7	AAV treatment	6	WT +GFP 3M3F / Mut +GFP 2M4F / WT +BDNF 4M2F / Mut +BDNF 5M1F	38-41
S1A-B	*in vitro* pull-downs	3	n.a.	n.a.
S1C-D	lumbar DRG western blots	5-6	WT 4M1F / Mut 3M3F	82-143
S1E-H	*Gars*$^{\Delta ETAQ/+}$ in vivo transport	6	WT 3M3F / Mut 5M1F	30-38
S1I-L	CMT2D in vivo transport	6	C201R 3M3F / ΔETAQ 5M1F	30-38
S2	muscle western blots	3-4	TA WT 1M/2F Mut 1M/2F MGC WT 1M/2F Mut 2M/2F	93

S3A-B	WT NMJ immunofluorescence	3	3M0F	132-140
S3C-G	WT muscle western blots	4-6	0M4-6F	77-83
S4	spinal cord motor neurons	5	WT 4M1F Mut 0M5F	104
S5	tibialis anterior fibre typing	6	WT 4M2F / Mut 2M4F	87-92
S6	sciatic nerve western blots	7	WT +vehicle 4M3F / Mut +vehicle 5M2F / WT +BDNF 4M3F / Mut +BDNF 2M5F	28-36
S7A-E	WT in vivo transport (+refametinib)	5	+DMSO 0M5F / +refametinib 0M5F	48-54
S7F-J	WT in vivo transport (+anti-BDNF)	6	+IgY 4M2F / +anti-BDNF 4M2F	32-34
S8A-D	WT in vivo transport	6-12	+vehicle 3M3F / +BDNF 3M3F	33-35
			+vehicle 3M3F / +BDNF 3M3F	90-97
			+vehicle 0M6F / +BDNF 0M12F	391-426
S8E-H	*Gars*$^{C201R/+}$ in vivo transport	6	+vehicle Mut 3M3F / +BDNF 3M3F	33-35
			+vehicle Mut 3M3F / +BDNF 3M3F	90-97
			+vehicle Mut 6M0F / +BDNF 6M0F	391-426
S9A-C	tissue western blots and muscle immunofluorescence (AAV)	3	3M0F	30 and 82
S9D-G	muscle western blots (AAV)	3	5.0×10^{10} 2M1F / 1.0×10^{11} 1M2F / 2.0×10^{11} 3M0F	23

S9H-J	muscle western blots and immunofluorescence (AAV)	6	WT GFP 3M3F / Mut GFP 2M4F / WT BDNF 4M2F / Mut BDNF 5M1F	38-41

Supplemental Table 2. Primary antibodies used in this study. *BDNF*, brain-derived neurotrophic factor; *ChAT*, choline acetyltransferase; *CHRNA1*, cholinergic receptor nicotinic alpha 1; *CREB*, cAMP response element-binding; *CST*, Cell Signaling Technology; *DSHB*, Developmental Studies Hybridoma Bank; *ERK*, extracellular signal-regulated kinase; *GAPDH*, glyceraldehyde-3-phosphate dehydrogenase; *GDNF*, glial cell line-derived neurotrophic factor; *GFP*, green fluorescent protein; *GlyRS*, glycyl-tRNA synthetase; *Gt*, goat; *HRP*, horseradish peroxidase; *IF*, immunofluorescence; *IgG/G1/G2b/M*, immunoglobulin G/G1/G2b/M; *MHC*, myosin heavy chain; *Ms*, mouse; *NF*, neurofilament; *PLC*, phospholipase C; *Rb*, rabbit; *RILP*, Rab-interacting lysosomal protein; *RRID*, Research Resource Identifier; *R&D*, R&D Systems; *Sp.*, species; SV, synaptic vesicle; *TDP-43*, TAR DNA-binding protein 43; *TrkB*, tropomyosin receptor kinase B; *WB*, western blotting.

**Target**	**Sp.**	**Clonality**	**Company /Reference**	**Catalogue #**	**RRID**	**IF dilution**	**WB dilution**
AKT	Rb	Poly	CST	9272	AB_329827	n/a	1:1,000
p-AKT (T308)	Rb	Poly	CST	9275	AB_329828	n/a	1:1,000
BDNF	Rb	Poly	Alomone	ANT-010	AB_2039756	1:300	1:500
BDNF	Ch	Poly	R&D	AF248	AB_355275	n/a	n/a
ChAT	Gt	Poly	Chemicon	AB144P	AB_2079751	1:250	n/a
CHRNA1	Rb	Poly	Alomone	ANC-001	AB_10613115	n/a	1:500
CREB	Rb	Mono	CST	9197	AB_331277	1:500	n/a
p-CREB (S133)	Rb	Mono	Abcam	ab32096	AB_731734	1:250	n/a
ERK1/2	Rb	Poly	CST	9102	AB_330744	n/a	1:1,000
p-ERK1/2 (T202/T204)	Rb	Poly	CST	9101	AB_331646	n/a	1:500
GAPDH	Ms	Mono	Merck	MAB374	AB_2107445	n/a	1:5,000
GDNF	Rb	Mono	Abcam	ab176564	n/a	n/a	1:500

GFP	Ms	Mono	Santa Cruz	sc-9996	AB_627695	n/a	1:1,000
GlyRS	Rb	Poly	Abcam	ab42905	AB_732519	n/a	1:2,000
Hook1	Rb	Mono	Abcam	ab150397	n/a	n/a	1:1,000
Human IgG Fc (HRP)	Gt	Poly	Abcam	ab98624	AB_10673832	n/a	1:10,000
laminin	Rb	Poly	Sigma	L9393	AB_477163	1:250	n/a
MHC type I	Ms	Mono (IgG2b)	DSHB	BA-D5	AB_2235587	1:100	n/a
MHC type IIa	Ms	Mono (IgG1)	DSHB	SC-71	AB_2147165	1:100	n/a
MHC type IIb	Ms	Mono (IgM)	DSHB	BF-F3	AB_2266724	1:100	n/a
neurofilament (2H3)	Ms	Mono	DSHB	2H3	AB_531793	1:250	n/a
NF200	Ms	Mono	Sigma	N0142	AB_477257	n/a	1:1,000
peripherin	Rb	Poly	Chemicon	AB1530	AB_90725	n/a	1:1,000
PLCγ1	Rb	Poly	CST	2822	AB_2163702	n/a	1:1,000
p-PLCγ1 (Y783)	Rb	Poly	CST	2821	AB_330855	n/a	1:1,000
RILP	Rb	Poly	Abcam	ab140188	n/a	n/a	1:1,000
Snapin	Rb	Poly	(5)	n/a	n/a	n/a	1:1,000
SV2 (pan)	Ms	Mono	DSHB	SV2	AB_2315387	1:50	n/a
TDP-43	Ms	Mono	Abcam	ab104223	AB_10710019	1:1,000	n/a
TrkB	Rb	Poly	Merck	07-225	AB_310445	1:200	1:500
α-tubulin	Ms	Mono	CST	3873	AB_1904178	n/a	1:2,000
V5	Ms	Mouse	Thermo Fisher	R960-25	AB_2556564	n/a	1:5,000

Supplemental Table 3. Secondary antibodies used in this study. *Dk*, donkey; *Gt*, goat; *IgG/G1/G2b/M*, immunoglobulin G/G1/G2b/M; *HRP*, horseradish peroxidase; *Ms*, mouse; *Rb*, rabbit; *RRID*, Research Resource Identifier; *Sp.*, species.

Target	Sp.	Conjugate	Company	Catalogue #	RRID	Dilution
Gt IgG	Dk	AlexaFluor555	Life Technologies	A-21432	AB_2535853	1:250-500
Ms IgG	Dk	AlexaFluor488	Life Technologies	A-21202	AB_141607	1:250-500
Ms IgG	Dk	AlexaFluor555	Life Technologies	A-31570	AB_2536180	1:250-500
Ms IgG	Dk	AlexaFluor647	Life Technologies	A-31571	AB_162542	1:250-500
Ms IgG	Gt	HRP	Sigma	AP308P	AB_11215796	1:5,000
Ms IgG1	Gt	AlexaFluor647	Life Technologies	A-21240	AB_141658	1:500
Ms IgG2b	Gt	AlexaFluor488	Life Technologies	A-21141	AB_141626	1:500
Ms IgM	Gt	AlexaFluor568	Life Technologies	A-21043	AB_2535712	1:500
Rb IgG	Dk	AlexaFluor488	Life Technologies	A-21206	AB_2535792	1:250-500
Rb IgG	Dk	AlexaFluor555	Life Technologies	A-31572	AB_162543	1:250-500
Rb IgG	Dk	HRP	Bio-Rad	1706515	AB_11125142	1:5,000
Rb IgG	Gt	AlexaFluor405	Life Technologies	A-31556	AB_221605	1:500

Supplemental Table 4. Sequences used to create AAV plasmids. *BDNF*, brain-derived neurotrophic factor; *eGFP*, enhanced green fluorescent protein.

Element	Length (bp)	Sequence
Adapted *tMCK* promoter	745	AGATCGGAATTCGCCCTTAAGCTAGCCACTACGGGTCTAGGCTGCCC ATGTAAGGAGGCAAGGCCTGGGGACACCCGAGATGCCTGGTTATAAT TAACCCCAACACCTGCTGCCCCCCCCCCCCCAACACCTGCTGCCTGAG CCTGAGCGGTTACCCCACCCCGGTGCCTGGGTCTTAGGCTCTGTACAC CATGGAGGAGAAGCTCGCTCTAAAAATAACCCTGTCCCTGGTGGGCC CACTACGGGTCTAGGCTGCCCATGTAAGGAGGCAAGGCCTGGGGACA CCCGAGATGCCTGGTTATAATTAACCCCAACACCTGCTGCCCCCCCCC CCCCAACACCTGCTGCCTGAGCCTGAGCGGTTACCCCACCCCGGTGCC TGGGTCTTAGGCTCTGTACACCATGGAGGAGAAGCTCGCTCTAAAAA TAACCCTGTCCCTGGTGGGCCACTACGGGTCTAGGCTGCCCATGTAAG GAGGCAAGGCCTGGGGACACCCGAGATGCCTGGTTATAATTAACCCC AACACCTGCTGCCCCCCCCCCCCCAACACCTGCTGCCTGAGCCTGAGC GGTTACCCCACCCCGGTGCCTGGGTCTTAGGCTCTGTACACCATGGAG GAGAAGCTCGCTCTAAAAATAACCCTGTCCCTGGTGGGCCCCTCCCTG GGGACAGCCCCTCCTGGCTAGTCACACCCTGTAGGCTCCTCTATATAA CCCAGGGGCACAGGGGCTGCCCCCGGGTCAC
human *BDNF* (pre-pro form)	744	ATGACCATCCTTTTCCTTACTATGGTTATTTCATACTTTGGTTGCATGA AGGCTGCCCCCATGAAAGAAGCAAACATCCGAGGACAAGGTGGCTTG GCCTACCCAGGTGTGCGGACCCATGGGACTCTGGAGAGCGTGAATGG GCCCAAGGCAGGTTCAAGAGGCTTGACATCATTGGCTGACACTTTCG AACACGTGATAGAAGAGCTGTTGGATGAGGACCAGAAAGTTCGGCCC AATGAAGAAAACAATAAGGACGCAGACTTGTACACGTCCAGGGTGAT GCTCAGTAGTCAAGTGCCTTTGGAGCCTCCTCTTCTCTTTCTGCTGGA GGAATACAAAAATTACCTAGATGCTGCAAACATGTCCATGAGGGTCC GGCGCCACTCTGACCCTGCCCGCCGAGGGGAGCTGAGCGTGTGTGAC AGTATTAGTGAGTGGGTAACGGCGGCAGACAAAAAGACTGCAGTGG ACATGTCGGGCGGGACGGTCACAGTCCTTGAAAAGGTCCCTGTATCA AAAGGCCAACTGAAGCAATACTTCTACGAGACCAAGTGCAATCCCAT GGGTTACACAAAAGAAGGCTGCAGGGGCATAGACAAAAGGCATTGG AACTCCCAGTGCCGAACTACCCAGTCGTACGTGCGGGCCCTTACCAT GGATAGCAAAAAGAGAATTGGCTGGCGATTCATAAGGATAGACACTT CTTGTGTATGTACATTGACCATTAAAAGGGGAAGATAG
eGFP	714	GTCAGCAAGGGCGAGGAACTGTTCACCGGGGTGGTGCCCATCCTGGT CGAGCTGGACGGCGACGTAAACGGCCACAAGTTCAGCGTGTCCGGCG AGGGCGAGGGCGATGCCACCTACGGCAAGCTGACCCTGAAGTTCATC TGTACCACCGGCAAGCTGCCCGTGCCCTGGCCCACCCTCGTGACCACC CTGACCTACGGCGTGCAATGCTTCAGCCGCTACCCCGACCACATGAA GCAGCACGACTTCTTCAAGTCCGCCATGCCCGAAGGCTACGTCCAGG AGCGCACCATCTTCTTCAAGGACGACGGCAACTACAAGACCCGCGCC GAGGTGAAGTTCGAGGGCGACACCCTGGTGAACCGCATCGAGCTGAA GGGCATCGACTTCAAGGAGGACGGCAACATCCTGGGGCACAAGCTGG AGTACAACTACAACAGCCACAACGTCTATATCATGGCCGACAAGCAG AAGAACGGCATCAAGGTGAACTTCAAGATCCGCCACAACATCGAGGA CGGCAGCGTGCAACTCGCCGACCACTACCAGCAGAACACCCCCATCG GCGACGGCCCCGTGCTGCTGCCCGACAACCACTACCTGAGCACCCAG TCCGCCCTGAGCAAAGACCCCAACGAGAAGCGCGATCACATGGTCCT GCTGGAGTTCGTGACCGCCGCCGGGATCACTCTCGGCATGGACGAGC TGTACAAG

Supplementary References

1. Sleigh JN, et al. Trk receptor signaling and sensory neuron fate are perturbed in human neuropathy caused by *Gars* mutations. *Proc Natl Acad Sci U S A.* 2017;114(16):E3324-E3333.
2. Sleigh JN, et al. Altered sensory neuron development in CMT2D mice is site-specific and linked to increased GlyRS levels. *Front Cell Neurosci.* 2020;14:232.
3. Sleigh JN, et al. Developmental demands contribute to early neuromuscular degeneration in CMT2D mice. *Cell Death Dis.* 2020;11(7):564.
4. Chao MV. Neurotrophins and their receptors: A convergence point for many signalling pathways. *Nat Rev Neurosci.* 2003;4(4):299-309.5.
5. Granata A, et al. The dystonia-associated protein torsinA modulates synaptic vesicle recycling. *J Biol Chem.* 2008;283(12):7568-7579.
